# CPNE5 overexpression inhibits cardiomyocytes apoptosis by promoting the degradation of FAS receptor

**DOI:** 10.1016/j.isci.2025.113302

**Published:** 2025-08-06

**Authors:** Tingting Zhao, Yangjinming Bai, Yudong Fei, Zhixing Wei, Pengcheng Yao, Qianji Che, Yichao Zhang, Ji Yan, Kaiyan Chen, Zhengyang Wu, Junhao Qiu, Yuepeng Wang, Wei Li, Qian Wang, Yigang Li

**Affiliations:** 1 Department of Cardiology, Xinhua Hospital Affiliated to Shanghai Jiao Tong University School of Medicine, 1665 Kongjiang Road, Shanghai 200092, China

**Keywords:** Rodent cardiology, Rodent molecular biology, Biological sciences

## Abstract

CPNE5, a member of the Copine family, is characterized by its membrane-binding properties and functions as a regulatory modulator of intracellular signaling through the spatial redistribution of interacting protein partners. Emerging evidence has demonstrated that CPNE3 exerts cardioprotective effects via anti-apoptotic activity in myocardial ischemia-reperfusion injury models. However, the functional role of CPNE5 in cardiac pathology remains unclear. In this study, the cardiac-specific overexpression of CPNE5 in mice improved cardiac function, reduced cellular apoptosis, and attenuated cardiac fibrosis in both transverse aortic constriction and ischemia-reperfusion models. Conversely, CPNE5 knockout mice exhibited opposite pathological phenotypes. Mechanistic studies revealed that CPNE5 retains FAS within the endoplasmic reticulum and promotes its degradation through the ER-phagy pathway. This process involves CPNE5’s interaction with the autophagy marker LC3 and CALCOCO1, a key receptor in the ER-lysosome-associated degradation (ERLAD) pathway. Collectively, these findings indicate that CPNE5 overexpression protects cardiomyocytes against FASL-induced apoptosis under stress and ischemic conditions.

## Highlights

• CPNE5 expression is elevated in TAC and I/R induced heart failure models
• CPNE5 can improve cardiac function and inhibit cardiomyocytes apoptosis in TAC and I/R mice
• CPNE5 interacts with FAS and promotes the degradation of FAS protein through the ERLAD pathway
• Overexpression of CPNE5 inhibited FASL induced apoptosis of neonatal cardiomyocytes

## Graphical abstract

**Figure:**
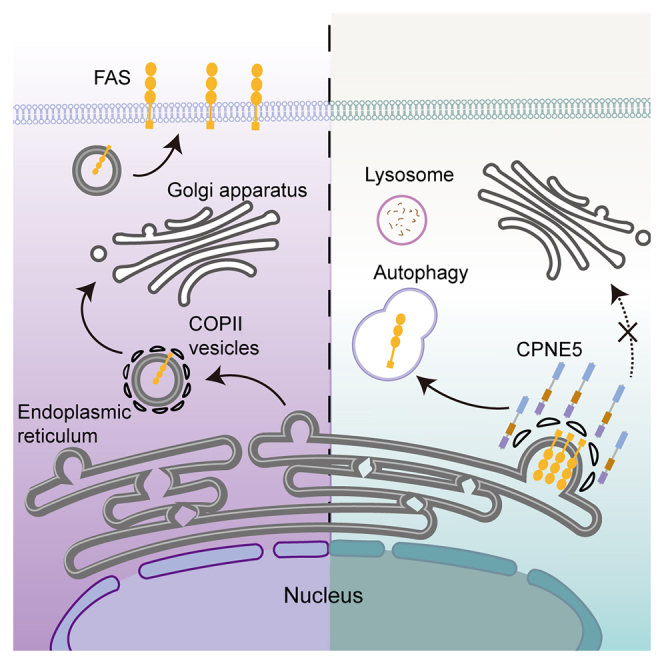

## Introduction

Heart failure (HF) is the result of long-term exposure of the heart to chronic stresses or injuries, such as pressure or volume overload (such as hypertension, valvular heart disease), myocardial infarction or ischemia, or genetic disorders.^1^^,^^2^ Both in the chronically stimulated heart subjected to long-term pressure load and in the acutely stimulated heart due to ischemia, myocardial cell death is an irreversible pathological process leading to the deterioration of cardiac function.^3^^,^^4^ Because of the non-regenerative nature of cardiomyocytes in adulthood, inhibiting cardiomyocyte death in heart failure has become an important target for the treatment of heart failure.^5^^,^^6^^,^^7^

Apoptosis is the main form of programmed death in cardiomyocytes. It is mainly initiated through two pathways: the extrinsic pathway (also known as the death receptor-mediated pathway) and the intrinsic pathway (also called the mitochondria-mediated pathway).^8^ FAS is one of the transmembrane receptors that executes the extrinsic apoptotic pathway. It belongs to a member of the tumor necrosis factor (TNF) gene superfamily and exists constitutively in muscle cells.^9^

In type I cells, the binding of FASL (FAS ligand) to FAS initiates a ceramide dependent formation of the DISC (death-inducing signaling complex) and Caspase8-mediated clustering of FAS at the plasma membrane. This is followed by the internalization of these multimeric receptor-DISC complexes. In contrast, hepatocytes, which are type II cells, predominantly store FAS intracellularly under resting conditions, with only a small number of “sentinel” FAS receptors present on the plasma membrane. Nonetheless, the activation of these receptors by FASL is sufficient to induce Caspase8 dependent endosomal acidification and ceramide mediated trafficking of intracellularly stored FAS to the plasma membrane, thereby amplifying FAS activation.^10^^,^^11^ Moreover, since DISC formation occurs after endocytosis of FAS, it has been shown that FAS mediated apoptosis can be inhibited by decreasing its recycling process through endosomes back to the plasma membrane.^12^ It is suggested that the regulation of the intracellular FAS transport pathway may be an effective strategy to prevent FAS-mediated apoptosis.^13^^,^^14^

CPNE5 is a member of the copine family (CPNEs). Nine CPNE proteins have been identified, and eight are found in mammals (CPNE1-8). CPNEs are soluble membrane proteins containing two C2 domains and one A domain.^15^ The C2 domain has a calcium-dependent phospholipid binding function. A domain plays a role in protein binding.^16^ Previous research has indicated that mutations in the Ca^2+^ binding sites within the C2 domain of CPNE1 and CPNE6 can impair their localization to the membrane.^17^^,^^18^ CPNE1 binds to the annexin through the C2 domain on the lysosomal membrane under Ca^2+^ stimulation and affects the fusion of lysosomes and endosomes to regulate autophagy.^19^^,^^20^ CPNE3 overexpression inhibits the release of inflammatory factors induced by hypoxia/reoxygenation in cardiomyocytes and reduces cell apoptosis by activating RACK1 expression.^21^ However, the function of CPNE5 in HF remains unknown.

In the present study, we found that the overexpression of CPNE5 could inhibit FAS protein expression, improve cardiac function, and inhibit cardiomyocytes apoptosis in mouse HF models induced by transverse aortic constriction (TAC) or ischemia/reperfusion (I/R). The mechanism of CPNE5 induced FAS reduction was further explored at the level of primary neonatal mouse cardiomyocytes. We observed that CPNE5 preconditioning led to the retention of FAS in the endoplasmic reticulum (ER) and further facilitated its degradation through the endoplasmic reticulum lysosomal-associated degradation (ERLAD) pathway, ultimately rendering cardiomyocytes resistant to FASL-induced apoptosis. Taken together, our findings provide evidence for strategies to interfere with FAS localization to the cell membrane during the early secretory phase, which could serve as molecular targets for the treatment of heart failure.

## Results

### CPNE5 expression is upregulated in the mouse transverse aortic constriction and ischemia-reperfusion models

**Figure 1:**
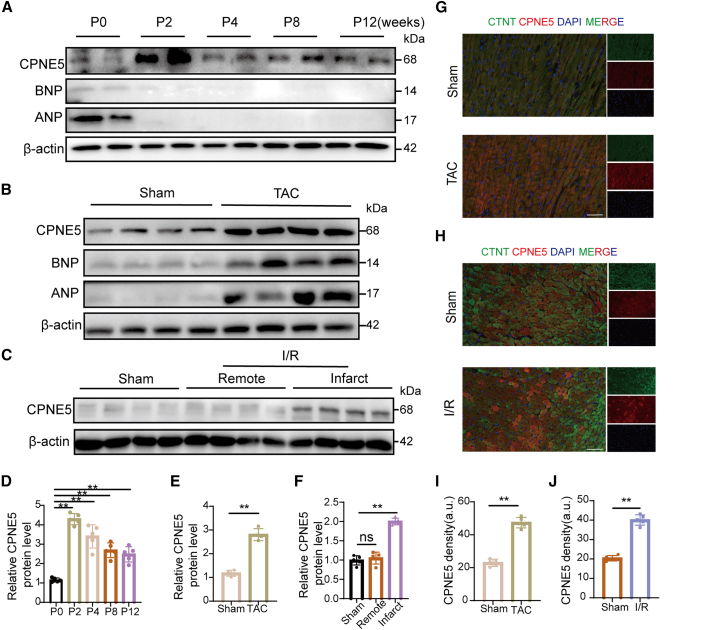
CPNE5 is associated with heart development and heart failure (A) Western blot showed the expression of CPNE5, BNP, and ANP protein levels in heart tissue at postnatal (P) 0, 2, 4, 8, 12 weeks, respectively (*n* = 5). β-actin was used as the internal control. (B) The expression of CPNE5, BNP, and ANP protein levels in heart tissues from Sham (*n* = 4) vs. TAC (*n* = 4). (C) CPNE5 protein levels in heart tissue from mice subjected to Sham (*n* = 5) or 1 week after I/R operation (*n* = 5). (D–F) Quantification of CPNE5 protein level of (A, B, C), normalized to β-actin. (G and H) Representative immunofluorescence staining for CPNE5 (red) in TAC (G, *n* = 5) and I/R (H, *n* = 5) myocardium (green), and the nucleus is blue (bar = 50 μm). (I and J) Showing relative CPNE5 immunofluorescence intensity in group TAC (G) or I/R (H). All data are presented as mean ± SD; Unpaired two-tailed Student’s t test (two groups) and one-way ANOVA with Dunnett’s post hoc test (more than two groups) were used to determine statistical significance of experimental data. ∗∗, *p* < 0.01; ns, not significantly different.

To explore the role of CPNE5 in the heart, we first examined the change of CPNE5 in the mouse heart under physiological conditions. We found that the expression of fetal genes ANP and BNP decreased with aging, which indicated the gradual maturation of the heart,^22^ and the protein expression of CPNE5 increased after birth (Figures 1A and 1D), suggesting that CPNE5 may be involved in the regulation of cardiac maturation during postnatal development in mice. Furthermore, the expression of CPNE5 was also significantly increased in chronic heart failure induced by TAC (Figures 1B and 1E) and acute heart failure induced by I/R (Figures 1C and 1F). Immunofluorescence staining further confirmed the increase of CPNE5 protein level in the failed myocardium (Figures 1G–1J). These findings imply that CPNE5 plays an important role in the maintenance of the basal function of cardiomyocytes and in the response to pathological factors.

### Cardiac-specific overexpression of CPNE5 improved cardiac function in the transverse aortic constriction and ischemia-reperfusion model

**Figure 2:**
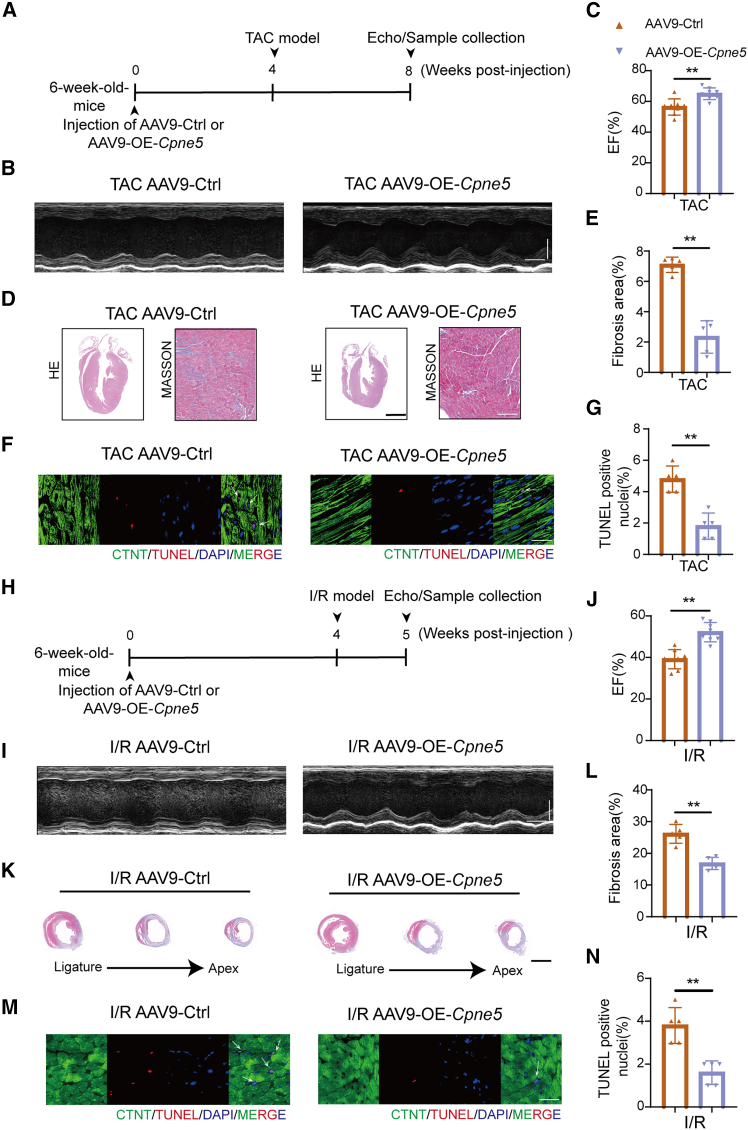
Overexpression of CPNE5 prevents cardiac remodeling and dysfunction after pathological stress (A and H) A schematic representation showing AAV9-Ctrl and AAV9-OE-*Cpne5* mice subjected to 4 weeks of TAC (A) or 1 week of I/R (H) operation. (B and I) Representative M-mode recordings of echocardiography of AAV9-Ctrl and AAV9-OE-*Cpne5* mice subjected to TAC (B) or I/R (I), horizontal bar = 100 ms, vertical bar = 2 mm. (C and J) Quantitative analyses of echocardiographic measurements performed after TAC (C) or I/R (J) operation (*n* = 8). (D and K) Histological sections stained with H&E (D, bar = 2 mm) and Masson’s trichrome (D, bar = 100 μm; K, bar = 2 mm) in mice of AAV9-Ctrl and AAV9-OE-*Cpne5* mice 4 weeks after TAC (D) or 1 week after I/R (K) operation. (E and L) Quantification of cardiac fibrosis area in (D) and (K) (*n* = 5). (F and M) Representative heart sections for TAC (F) and I/R (M) groups show immunofluorescence of TUNEL (red, arrows), DAPI (blue, for nuclei), and cardiac troponin (green; bar = 25 μm). (G and N) Statistical analysis of TUNEL-positive cells in (F) and (M) (*n* = 5). All data are presented as mean ± SD; Unpaired two-tailed Student’s t test (two groups) was used to determine statistical significance of experimental data. ∗∗*p* < 0.01.

To establish a functional role of CPNE5 in the development of HF, we constructed one adeno-associated virus (AAV9-OE-*Cpne5*) that specifically overexpresses CPNE5 in cardiac myocytes (Figure S1A). Successful overexpression of CPNE5 was confirmed by western blot (Figure 6E) and RT-qPCR (Figure S1B). CPNE5 overexpression did not affect baseline cardiac contractile function or gross morphology in mice (Figures S1C–S1E). Mice were delivered with AAV9-Ctrl or AAV9-OE-*Cpne5* 4 weeks prior to TAC or I/R procedure (Figures 2A and 2H). At 4 weeks after TAC, CPNE5 overexpression improved cardiac systolic function (Figures 2B and 2C) and inhibited cardiac fibrosis (Figures 2D and 2E). In accordance with myocardial fibrosis, we detected substantially increased apoptosis in control mice (AAV9-Ctrl) but not in CPNE5 overexpressing mice (AAV9-OE-*Cpne5*) after TAC (Figures 2F and 2G). Meanwhile, histological analysis using HE staining revealed that TAC-induced cardiac dilatation and hypertrophy were attenuated in CPNE5 overexpressing mice compared with control mice. To further characterize the effects of CPNE5 in another clinically relevant model of heart failure,^23^ we subjected mice to I/R surgery (Figure 2H). CPNE5 overexpression mice showed an increased cardiac ejection fraction (Figures 2I and 2J), a decreased area of cardiac fibrosis (Figures 2K and 2L), and a lower number of apoptotic cells (Figures 2M and 2N) compared with AAV9-Ctrl after 1 week of I/R. These findings suggest that CPNE5 plays a protective role in maintaining cardiac function and structure, mitigating the adverse effects of TAC and I/R.

### CPNE5 deficiency leads to exacerbated cardiac remodeling and dysfunction after pathological stress

**Figure 3:**
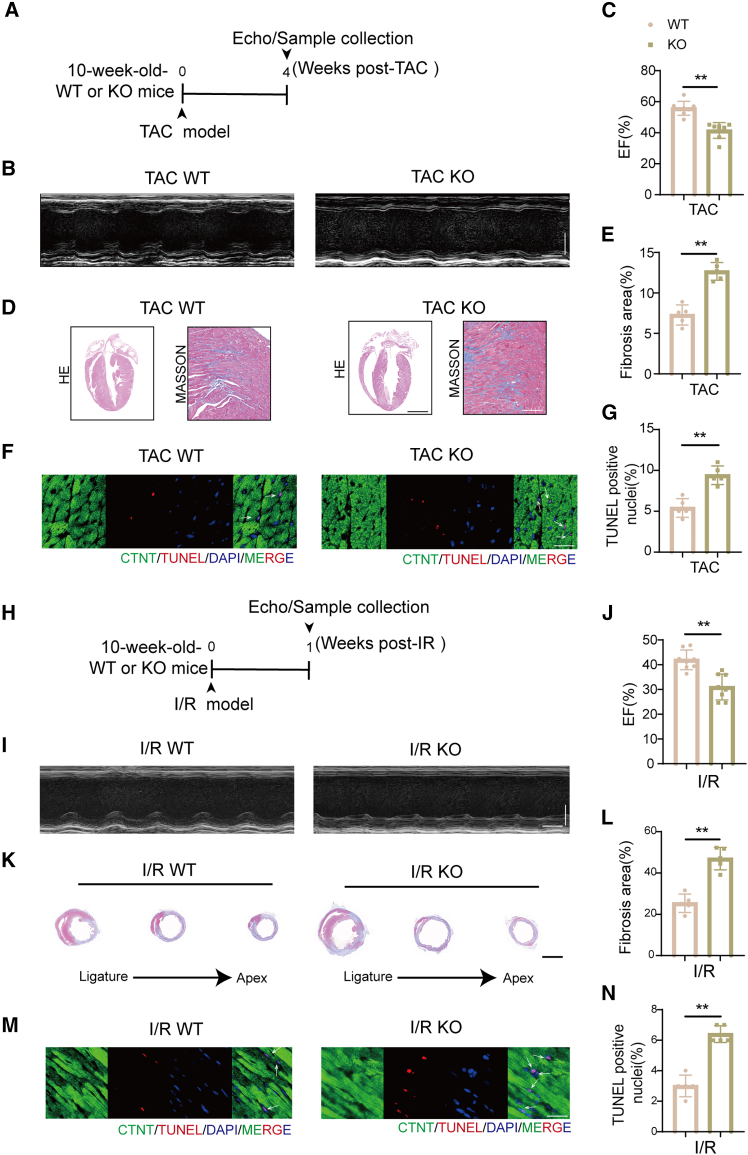
CPNE5 deficient mice show worsened cardiac function in the TAC and I/R model (A and H) A schematic representation shows WT and KO mice subjected to 4 weeks of TAC (A) or 1 week of I/R(H) operation. (B and I) Representative M-mode recordings of echocardiography of WT and KO mice subjected to TAC (B) or I/R (I), horizontal bar = 100 ms, vertical bar = 2 mm. (C and J) Quantitative analyses of echocardiographic measurements performed after TAC (C) or I/R (J) operation (*n* = 8). (D and K) Histological sections stained with H&E (D, bar = 2 mm) and Masson’s trichrome (D, bar = 100 μm; K, bar = 2 mm) in mice of WT and KO mice 4 weeks after TAC (D) or 1 week after I/R(K) operation (*n* = 5). (E and L) Quantification of cardiac fibrosis area in (D) and (K). (F and M) Representative heart sections for TAC (F) and I/R(M) groups show the immunofluorescence of TUNEL (red, arrows), DAPI (blue, for nuclei), and cardiac troponin (green; bar = 25 μm). (G and N) Statistical analysis of TUNEL-positive cells in (F) and (M) (*n* = 5). All data are presented as mean ± SD; unpaired two-tailed Student’s t test (two groups) was used to determine statistical significance of experimental data. ∗∗*p* < 0.01.

In order to fully elucidate the potential role of CPNE5 in stress conditions, the whole-body CPNE5 knockout mice were also utilized (Figure S2A), and the expression level of CPNE5 heterozygous mice was confirmed (Figure S2B). Under baseline conditions, the deletion of CPNE5 did not affect cardiac contractile function or gross morphology in mice (Figures S2C–S2E). WT and CPNE5 KO mice were then subjected to TAC (Figure 3A). Four weeks after TAC, CPNE5 KO mice exhibited severe cardiac dysfunction compared with WT mice. Echocardiography revealed marked reductions in ejection fraction (Figures 3B and 3C), indicating impaired cardiac performance. Moreover, histological analysis showed extensive fibrosis in CPNE5 KO hearts (Figures 3D and 3E), and the TUNEL positive cardiomyocytes were also notably increased in CPNE5 KO mice (Figures 3F and 3G).

Morphological assessment through HE staining indicated that CPNE5 depletion significantly aggravated TAC-induced cardiac chamber enlargement in comparison with WT mice. Consistent with previous findings, after one week of the I/R model, CPNE5 depletion (Figure 3H) led to marked cardiac dysfunction, characterized by a significant reduction in ejection fraction (Figures 3I and 3J), an expansion of fibrotic regions (Figures 3K and 3L), and increased cellular apoptosis (Figures 3M and 3N), compared with WT animals. Collectively, these data suggest that CPNE5 deficiency accentuates TAC or I/R elicited myocardial dysfunction and cardiomyocyte death.

### CPNE5 induced FAS degradation via lysosome-dependent pathway

**Figure 4:**
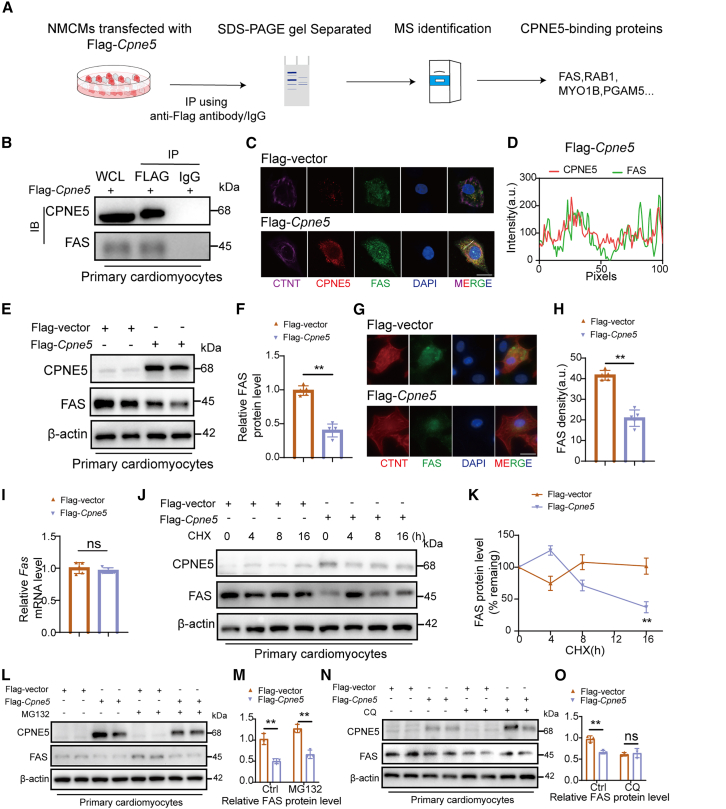
CPNE5 interacts with FAS and results in decreased FAS protein levels (A) Schematic diagram shows MS analysis workflow for identifying targets of CPNE5 in NMCMs. (B) IP analysis with anti-FLAG antibody and immunoblotting (IB) with antibodies of anti-Flag and anti-FAS, respectively, in NMCMs transfected with Flag-*Cpne5*. WCL: whole cell lysates. IgG was used as a negative control to rule out nonspecific interactions. (C) Immunofluorescence revealed the colocalization of CPNE5 (red) and FAS (green) in NMCMs (purple), the nucleus is blue, bar = 30 μm. (D) The intensity profiles of CPNE5 and FAS along the white dotted line in NMCMs (purple), which transfected with Flag-*Cpne5*. (E) Western blot analysis confirmed FAS protein level in CPNE5 overexpressing NMCMs. (F) Quantification FAS protein level of (E), normalized to β-actin (*n* = 5). (G) Representative Immunofluorescence staining for FAS (green) in Flag-vector and Flag-*Cpne5* NMCMs (red), the nucleus is blue, bar = 30 μm. (H) Relative FAS immunofluorescence intensity in (G) (*n* = 5). (I) RT-PCR analysis of relative *FAS* mRNA level in Flag-vector and Flag-*Cpne5* NMCMs (*n* = 5). (J and K) Western blot of FAS protein half-life in Flag-vector and Flag-*Cpne5* NMCMs treated with CHX. The quantification was shown in (K) (*n* = 3). (L and M) Western blot detected FAS protein level in Flag-vector and Flag-*Cpne5* NMCMs treated with MG132 (L), the quantification was shown in (M) (*n* = 3). (N and O) Western blot detected FAS protein level in Flag-vector and Flag-*Cpne5* NMCMs treated with CQ (N), the quantification was shown in (O) (*n* = 3). All data are presented as mean ± SD; Unpaired two-tailed Student’s t test (two groups) and one-way ANOVA with Dunnett’s post hoc test (more than two groups) were used to determine statistical significance of experimental data. ∗∗*p* < 0.01; ns, not significantly different.

Further studies are warranted to explore the underlying mechanisms and potential therapeutic applications of CPNE5 in HF management. Based on previous studies on the CPNE family,^16^ we propose that CPNE5 functions by interacting with other molecules to regulate the localization of target molecules. So, we overexpressed the Flag-*Cpne5* plasmid in NMCMs (Figure S3) and screened for CPNE5 binding proteins using immunoprecipitation-massspectrometry (IP-MS) (Figure 4A), and focused on genes involved in apoptosis signaling pathway. Among the precipitated proteins, we selected FAS to further validation. Because FAS is implicated in extrinsic apoptotic pathway which is a member of the tumor necrosis factor (TNF) gene superfamily. FASL is also a cell surface molecule belonging to the TNF family. By binding to its receptor FAS, FASL induces the oligomerization of FAS, and induces the recruitment of the Fas-associated death domain (FADD) to the cytoplasmic tail of FAS via its DD (death domain). The other end of FADD contains a DED (death effector domain) that allows the recruitment of Caspase8. Caspase8 induces apoptosis by activating downstream Caspase3. The results of IP-MS were further validated in primary NMCMs. We observed that exogenous Flag-CPNE5 interacts with FAS but not control IgG (Figure 4B). Consistently, immunofluorescence showed colocalization between Flag-CPNE5 and FAS in NMCMs (Figures 4C and 4D). The interaction between Flag-CPNE5 and FAS was also found in CPNE5 overexpressing mouse tissues (Figure S4). These results suggest an interaction between CPNE5 and FAS in cardiomyocytes under physiological condition, and suggest that CPNE5 may play a regulatory role in FAS mediated apoptosis.

To explore this possibility, we conducted a series of functional assays. We found that the FAS protein level declined after the overexpression of CPNE5 in NMCMs (Figures 4E and 4F). Immunofluorescence staining of NMCMs also showed a decrease in the expression level of FAS after the overexpression of CPNE5 (Figures 4G and 4H). Whereas the overexpression of CPNE5 did not change the mRNA level of FAS (Figure 4I), indicating that the CPNE5 regulation of FAS occurs post-transcriptionally. To explore the molecular mechanism of CPNE5 mediated FAS downregulation, we investigated the effect of CPNE5 on FAS protein stability. We found that the overexpression of CPNE5 promoted the degradation rate of FAS protein after the inhibition of protein synthesis by CHX in NMCMs (Figures 4J and 4K). Moreover, the treatment of lysosome inhibitor CQ (Figures 4N and 4O) rather than proteasome inhibitor MG132 (Figures 4L and 4M), abolished CPNE5 mediated FAS degradation, suggesting that CPNE5 promoted the degradation of FAS through a lysosome dependent pathway. All the findings above underscore the pivotal role of CPNE5 in modulating FAS levels via lysosomal degradation, offering a insight into the post-transcriptional regulation of FAS in cardiomyocytes. Further studies are required to clarify the precise molecular pathways through which CPNE5 facilitates FAS degradation.

### CPNE5 induced the endoplasmic reticulum retention of FAS and facilitated autophagy degradation

**Figure 5:**
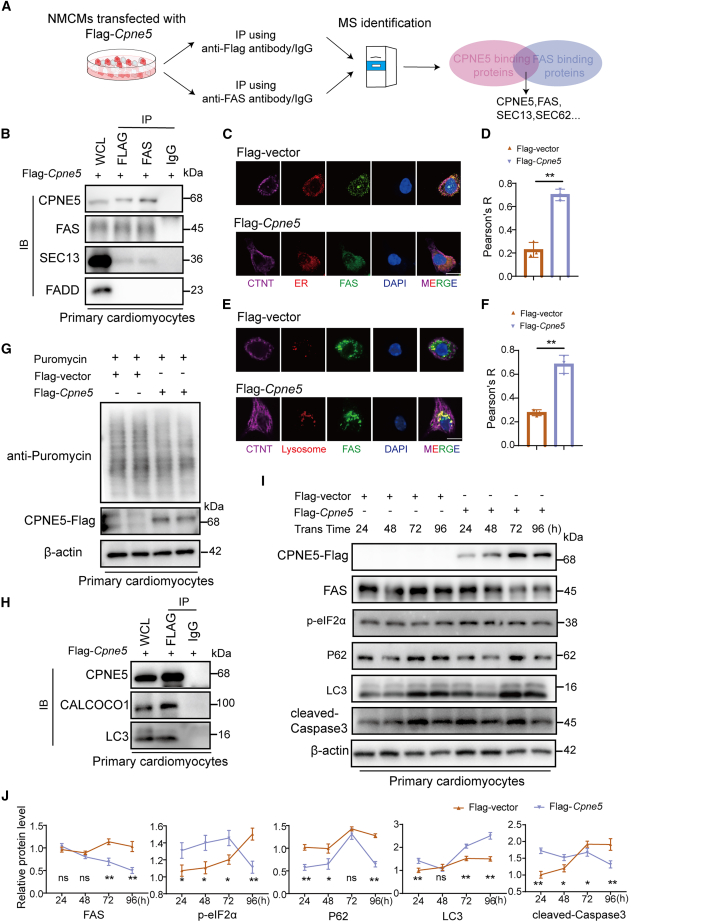
CPNE5 leads to FAS degradation via the ERLAD pathway (A) Schematic diagram shows MS analysis workflow for identifying targets of CPNE5 and FAS in NMCMs. (B) IP analysis with anti-FLAG and anti-FAS antibodies and IB with anti-bodies of CPNE5, FAS, SEC13, and FADD, respectively, in NMCMs transfected with Flag-*Cpne5*. WCL: whole cell lysates. IgG was used as a negative control to rule out nonspecific interactions. (C and D) Fluorescence confocal detected the localization of FAS (green) and ER marker (red) in NMCMs (purple), the nucleus is blue, bar = 30 μm, Pearson’s R value was analyzed by ImageJ (D) (*n* = 3). (E and F) Fluorescence confocal detected the localization of FAS (green) and lysosome marker (red) in NMCMs (purple), the nucleus is blue, bar = 30 μm, Pearson’s R value was analyzed by ImageJ (F) (*n* = 3). (G) Western blot analysis of peptides with puromycin in CPNE5 overexpressing NMCMs. (H) IP analysis with anti-FLAG antibody and IB with anti-bodies of CPNE5, CALCOCO1, and LC3, respectively, in NMCMs transfected with Flag-*Cpne5*. WCL: whole cell lysates. IgG was used as a negative control to rule out nonspecific interactions. (I and J) Western blot detected CPNE5, FAS, *p*-eiF2α, P62, LC3, cleaved-Caspase3 protein level in NMCMs (*n* = 3) at different times after transfected (trans) with Flag-vector and Flag-*Cpne5*. All data are presented as mean ± SD; unpaired two-tailed Student’s t test (two groups) and one-way ANOVA with Dunnett’s post hoc test (more than two groups) were used to determine statistical significance of experimental data. ∗∗*p* < 0.01; ∗*p* < 0.05; ns, not significantly different.

To further elucidate the mechanism behind the reduction of FAS protein, we conducted an IP assay using an FAS antibody in Flag-*Cpne5* overexpressing NMCMs. Subsequently, we identified the FAS interacting molecules in NMCMs following the overexpression of Flag-*Cpne5*, utilizing mass spectrometry. Furthermore, the molecules interacting with FAS were intersected (Figure 5A) with molecules that IP with Flag-*Cpne5* (Figure 4A). We discovered that CPNE5-interacting molecules were predominantly associated with the endoplasmic reticulum (ER). Considering that newly synthesized FAS will be sequentially transported to the ER and the Golgi apparatus for post-translational modification before being located on the cell membrane. Based on the findings of mass spectrometry, we hypothesized that CPNE5 regulated FAS transport from the ER to the Golgi apparatus. To validate this, SEC13 was selected from the cross-set of molecules interacting with both FAS and CPNE5. SEC13 is a subunit of the coat protein complex II (COPII), pivotal in vesicle formation. COPII-coated vesicles mediate the transport of cargo proteins from the exit sites of the ER toward the early Golgi complex.^24^ We conducted IP assays and found that SEC13 binds to both FAS and CPNE5 in NMCMs cells, which overexpressed Flag-*Cpne5* (Figure 5B), the addition of FASL did not affect their interaction (Figure S6A). This suggests that CPNE5 may modulate FAS transport from the ER to the Golgi apparatus. However, neither CPNE5 nor FAS interacts with FADD (Figure 5B), suggesting that the interaction between CPNE5 and FAS does not lead to FAS-mediated signaling activation. Further, we employed co-localization studies using confocal microscopy to visualize FAS distribution in CPNE5 overexpressed cells, revealing a notable accumulation of FAS within the ER (Figures 5C and 5D). This observation supports our hypothesis, indicating that CPNE5 potentially impedes FAS trafficking, thereby influencing its function to trigger apoptosis. Additionally, CPNE5 induced the expression of *p*-eIF2ɑ in a time-dependent manner (Figure 5I) coupled with the decline of the rate of protein synthesis (Figure 5G), suggesting CPNE5 induces the retention of FAS in the ER and promotes the occurrence of ER stress. Moreover, our data imply that CPNE5 mediated ER stress may activate unfolded protein response (UPR) pathways,^25^ potentially leading to cell survival or death depending on the duration and intensity of the stress (Figures 5I and 5J). As we can see, with the extension of CPNE5 overexpression time, the degree of apoptosis is gradually reduced, which may be related to the decrease of P62 and increase of LC3 mediated autophagy (Figures 5I and 5J). Similarly, we found that in the TAC model, CPNE5 inhibits the activation of Caspase3 in a dose-dependent manner (Figures S6C and S6D). We simultaneously discovered that CPNE5 interacts with both the endoplasmic reticulum autophagy receptor CALCOCO1^26^ and the autophagosome marker LC3 (Figure 5H), Additionally, FASL did not influence this phenomenon (Figure S6B). As shown in Figures 5E and 5F, increased colocalization between FAS and lysosome (Lamp1) under treatment with CPNE5. Based on our experimental observations, we hypothesized that the early upregulation of CPNE5 induces FAS retention in the ER lumen, triggering ER stress and subsequently promoting apoptotic signaling. To counteract this stress response, the unfolded protein response (UPR) pathway is activated, leading to *p*-eIF2ɑ mediated attenuation of protein synthesis to reduce ER load. As CPNE5 expression progressively increases, it interacts with both ER-phagy receptors (CALCOCO1) and LC3, thereby enhancing ER-phagy activity. This compensatory mechanism facilitates the clearance of accumulated proteins and alleviates ER stress-induced apoptosis.

### CPNE5 inhibited FAS mediated apoptosis pathway *in vivo* and *in vitro*

**Figure 6:**
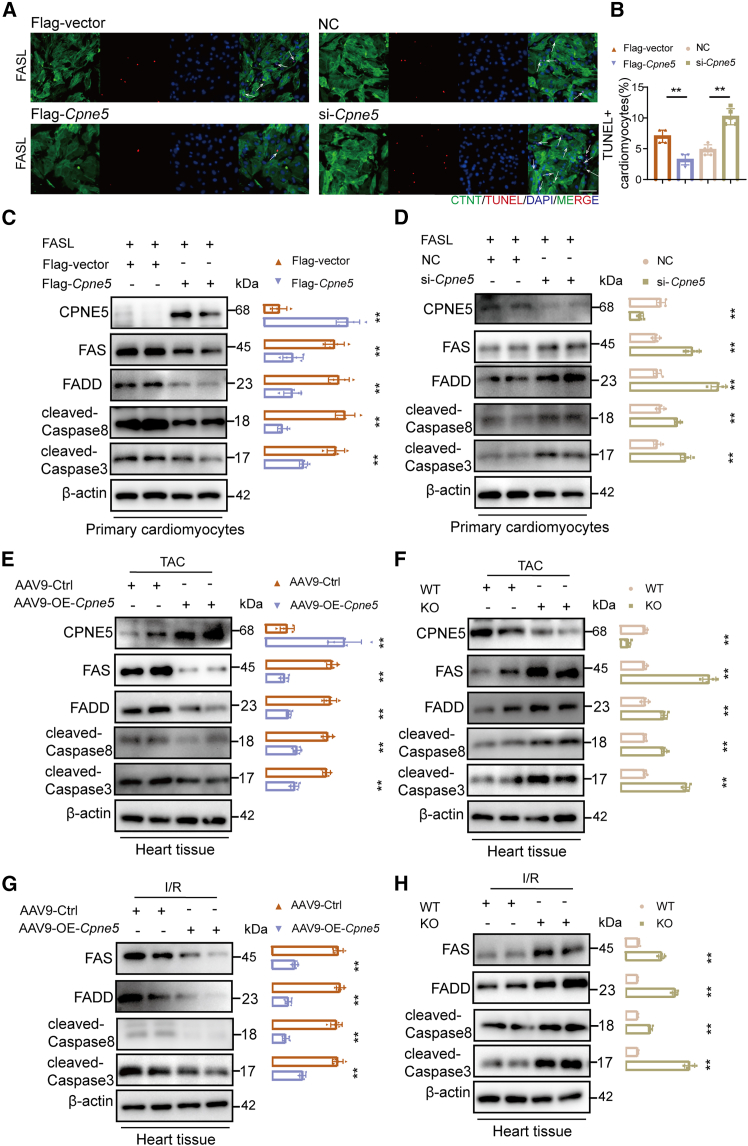
Overexpression of CPNE5 inhibits the activation of FAS receptor mediated apoptotic pathway *in vitro* and *in vivo* (A) Representative image showing immunofluorescence of TUNEL (red, arrows), DAPI (blue, for nuclei), and cardiac troponin (green; bar = 50 μm) in overexpression (Flag-*Cpne5*) or knockdown (si-*Cpne5*) CPNE5 NMCMs under FASL protein treatment. Flag-vector: empty vectors, NC: negative control. (B) Statistical analysis of TUNEL-positive cells in (A). (C and D) After treated with FASL protein, NMCMs overexpression CPNE5 (C) or knockdown CPNE5 group (D) were detected CPNE5, FAS, FADD, cleaved-Caspase8, and cleaved-Caspase3 protein levels by western blot. Bar graph on the right quantification protein level of (C) and (D), normalized to β-actin (*n* = 5). (E and G) After TAC (E) or I/R (G) model, AAV9-ctrl and AAV9-OE-*Cpne5* mice were detected for CPNE5, FAS, FADD, cleaved-Caspase8, and cleaved-Caspase3 protein levels by Western blot. Bar graph on the right quantification protein level of (E) and (G), normalized to β-actin (*n* = 5). (F and H) After TAC (F) or I/R (H) model, WT and KO mice were detected for CPNE5, FAS, FADD, cleaved-Caspase8, and cleaved-Caspase3 protein level by Western blot. Bar graph on the right quantification protein level of (F) and (H), normalized to β-actin (*n* = 5). All data are presented as mean ± SD; unpaired two-tailed Student’s t test (two groups) was used to determine statistical significance of experimental data. ∗∗*p* < 0.01.

Based on the above studies, we found that the overexpression of CPNE5 resulted in a decrease in FAS protein level. The alterations of FAS mediated apoptotic signaling pathway were further validated at both *in vitro* and *in vivo* levels. In NMCMs, the overexpression of CPNE5 inhibited FASL induced activation of the FAS signaling pathway (Figure 6C), which was consistent with TUNEL staining (Figures 6A and 6B). Conversely, knockdown of CPNE5 by using si RNA (Figure S7A) promoted FASL mediated apoptosis, as shown by the increase of cleaved-Caspase3 and Caspase8 and FADD (Figure 6D) and the increase of TUNE positive cells (Figures 6A and 6B). The *in vivo* experiments further corroborated these findings, demonstrating reduced cleaved-Caspase3 and cleaved-Caspase8 in CPNE5 overexpressing mice compared with controls after TAC and I/R (Figures 6E and 6G). However, CPNE5 KO mice showed increased activation of FAS downstream signaling pathways, showing the increased cleaved-Caspase3 and cleaved-Caspase8 and FADD (Figures 6F and 6H).

**Figure 7:**
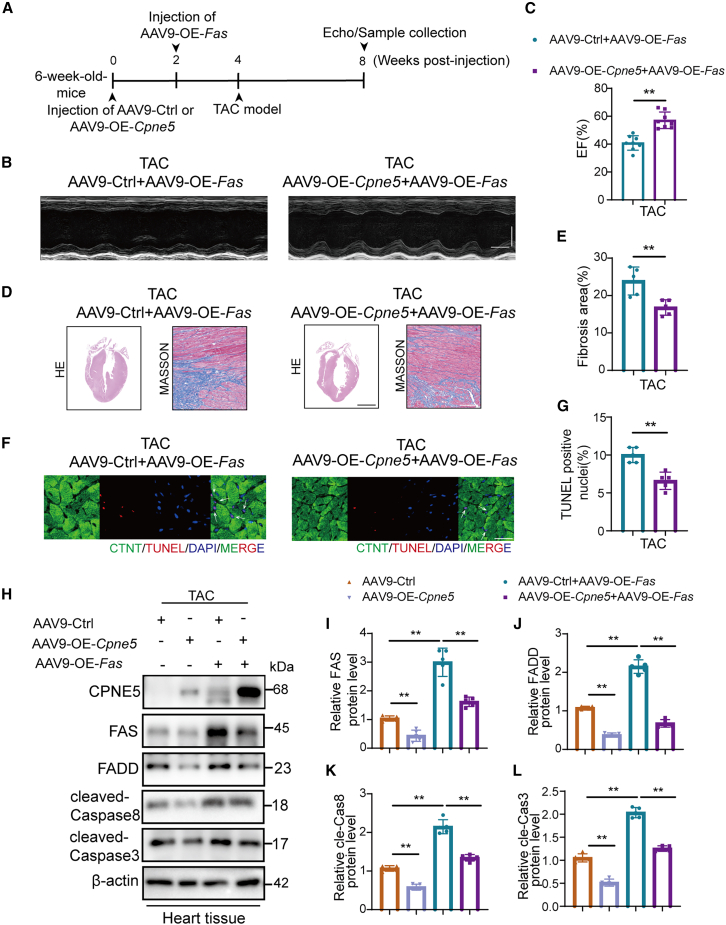
Overexpression of CPNE5 can partially reverse FAS mediated cardiac dysfunction in TAC mice (A) A schematic representation shows AAV9-Ctrl+AAV9-OE-*Fas* and AAV9-OE-*Cpne5*+AAV9-OE-*Fas* mice subjected to 4 weeks of TAC operation. (B) Representative M-mode recordings of echocardiography of AAV9-Ctrl+AAV9-OE-*Fas* and AAV9-OE-*Cpne5*+AAV9-OE-*Fas* mice subjected to TAC, horizontal bar = 100 ms, vertical bar = 2 mm. (C) Quantitative analyses of echocardiographic measurements performed after TAC operation (*n* = 8). (D) Histological sections stained with H&E (bar = 2 mm) and Masson’s trichrome (bar = 100 μm) in group AAV9-Ctrl+AAV9-OE-*Fas* and AAV9-OE-*Cpne5*+AAV9-OE-*Fas* mice after 4 weeks of TAC operation, (*n* = 5). (E) Quantification of cardiac fibrosis area in (D). (F) Representative heart sections for TAC groups show immunofluorescence of TUNEL (red, arrows), DAPI (blue, for nuclei), and cardiac troponin (green; bar = 25 μm). (G) Statistical analysis of TUNEL-positive cells in (F) (*n* = 5). (H) Western blot detected protein level in AAV9-Ctrl+AAV9-OE-*Fas* and AAV9-OE-*Cpne5*+AAV9-OE-*Fas* mice after TAC operation. (I–L) quantification FAS, FADD, cleaved-Caspase8, and cleaved-Caspase3 protein level of (H), normalized to β-actin (*n* = 5). All data are presented as mean ± SD; unpaired two-tailed Student’s t test (two groups) and one-way ANOVA with Dunnett’s post hoc test (more than two groups) were used to determine statistical significance of experimental data. ∗∗*p* < 0.01.

**Figure 8:**
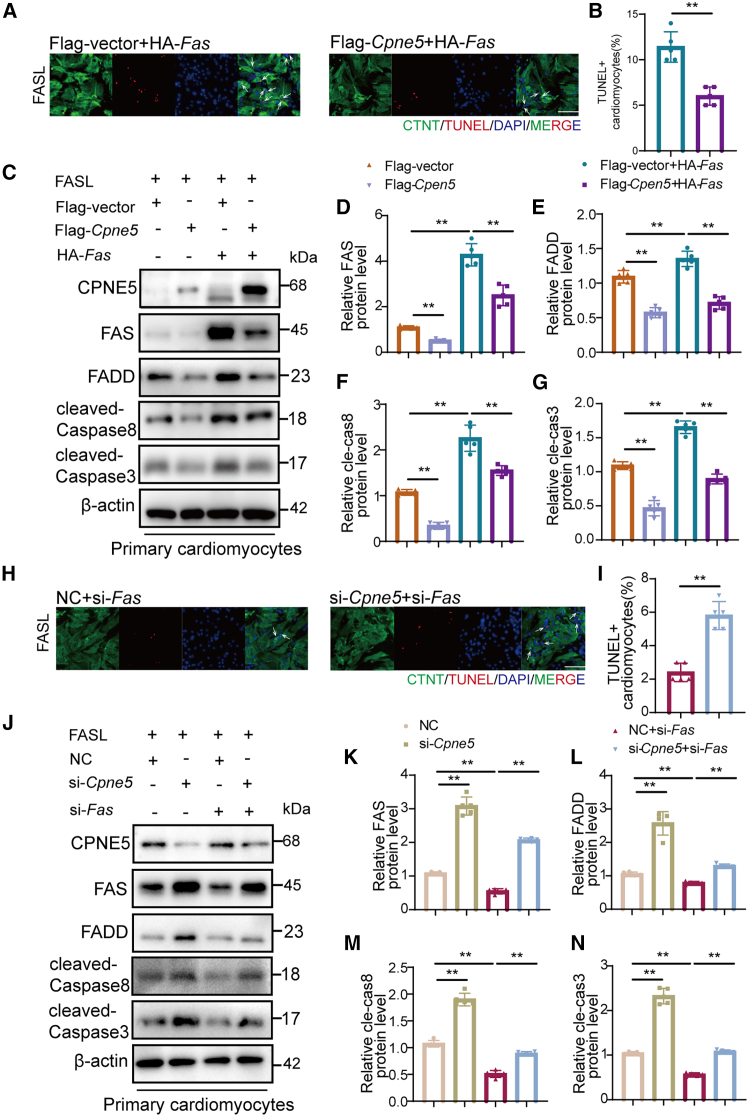
Overexpression of CPNE5 in cardiomyocytes reduces FAS mediated apoptosis of cardiomyocytes (A) Representative image showing immunofluorescence of TUNEL (red, arrows), DAPI (blue, for nuclei), and cardiac troponin (green; bar = 80 μm) in NMCMs, which infected with Flag-*Cpne5* and HA-*FAS* plasm id under FASL protein treatment. Flag-vector: empty vectors. (B) Statistical analysis of TUNEL-positive cells in (A). (C) Representative Western blot in cardiomyocytes infected with Flag-*Cpne5* and HA-*FAS* plasmid under FASL protein treatment. (D–G) Quantification of FAS, FADD, cleaved-Caspase8, and cleaved-Caspase3 protein level of (C), normalized to β-actin (*n* = 5). (H) Apoptotic cells were detected using the TUNEL assay after CPNE5 and FAS knockdown under FASL protein stimulation. NC: negative control. bar = 80 μm. (I) Statistical analysis of TUNEL-positive cells in (H). (J) After treated with FASL protein, NMCMs, which treated with si-*Cpne5* and si-*Fas* were detected CPNE5, FAS, FADD, cleaved-Caspase8, and cleaved-Caspase3 protein level by Western blot. (K–N) Quantification FAS, FADD, cleaved-Caspase8, and cleaved-Caspase3 protein levels of (J), normalized to β-actin (*n* = 5). All data are presented as mean ± SD; unpaired two-tailed Student’s t test (two groups) and one-way ANOVA with Dunnett’s post hoc test (more than two groups) were used to determine statistical significance of experimental data. ∗∗*p* < 0.01.

Finally we find the overexpression of CPNE5 attenuated AAV9-OE-*Fas* (Figure 7A) mediated cardiac dysfunction, including improved EF, decline fibrosis area and cardiomyocytes apoptosis at 4 weeks after TAC (Figures 7B–7G). Western blot analysis revealed that the overexpression of CPEN5 could partially inhibit the activation of apoptosis pathway caused by FAS overexpression (Figures 7H–7L). Cardiac-specific overexpression of FAS under basal conditions did not alter cardiac function or morphology in mice (Figure S8). We also found that the apoptosis induced by FAS overexpression *in vitro* could be partially inhibited by CPNE5 overexpression (Figures 8A–8G). Meanwhile, the decrease of apoptosis induced by FAS knockdown could be aggravated by CPNE5 knockdown (Figures 8H–8N). These results collectively indicate that CPNE5 plays a crucial role in modulating FAS mediated apoptosis, thereby influencing cardiac remodeling and function. Thus, CPNE5 overexpression could serve as a potential therapeutic strategy to mitigate apoptosis-driven cardiac damage.

## Discussion

With the aging trend, the incidence of coronary heart disease, hypertension, diabetes, obesity, and other chronic diseases is on the rise, resulting in the continued increase in the prevalence of heart failure.^27^ Despite remarkable progress in the study of its cellular mechanisms and treatment strategies, mechanistic knowledge is essential for developing therapeutics for patients with heart failure. In the present study, we found CPNE5 is significantly upregulated in response to TAC and I/R model. Premature overexpression of CPNE5 in cardiomyocytes with adeno-associated virus prevented cardiac dysfunction and apoptosis induced by TAC and I/R models. Conversely, CPNE5 whole body knockout mice showed deterioration of cardiac function after TAC and I/R, as evidenced by cardiac ejection fraction, fibrosis area, and cardiomyocytes apoptosis. Furthermore, we found that CPNE5 interacted with the death receptor FAS, altered the normal intracellular distribution of FAS, led to the retention of FAS in the ER, and promoted the degradation of the trapped FAS through the lysosome pathway.

Furthermore, we demonstrated that SEC13 interacts with both CPNE5 and FAS, suggesting a role of CPNE5 in COPII mediated FAS transport. Because we did not find a molecule that directly interacts with CPNE5, we cannot determine whether the retention of FAS in the ER is caused by the distribution characteristics of CPNE5 itself in the ER or by CPNE5 with the help of other molecules. Further studies should focus on the interaction between CPNE5 and other ER proteins, reveal the specific mechanism of CPNE5 in FAS transport.

Based on previous studies, we know that FAS mediated extrinsic apoptosis is mainly activated by FASL in TAC and I/R models.^3^^,^^4^^,^^28^ Therefore, in our study, by adding FASL protein to NMCMs to mimic FAS receptor activation *in vivo*, we found that the overexpression of CPNE5 in NMCMs resulted in the pre-down-regulation of FAS, which inhibited the FASL induced activation of the FAS signaling pathway on apoptosis execution molecule Caspase3. However, we found that CPNE5 had a protective effect on cardiomyocytes before the administration of FASL (Figures S7B and S7C). This may be due to the stress response of NMCMs subjected to mechanical shearing and trypsin digestion during isolation. So that NMCMs cannot completely mimic the basal state *in vivo*. At the same time, CPNE5 may have a pathway other than FAS to protect cardiomyocytes. Our finding of the role of CPNE5 in cardiomyocytes, together with previous findings of CPNE3 inhibiting apoptosis in the heart improves our understanding of the role of the CPNE family in the heart.^21^

In our study, we further found that CPNE5 caused the retention of FAS in the ER and further promoted the occurrence of ER stress. To alleviate ER stress, cells will initiate unfolded protein response pathways, such as eiF2α activation, to reduce translation efficiency.^29^

Moreover, the overloaded ER is cleared by ER autophagy mediated by SEC62, CALCOCO1.^26^^,^^30^ In our study, we also observed that the overexpression of CPNE5 decreased the selective autophagy receptor P62, and the increase of the autophagosome membrane structural protein LC3 indicated the autophagic flow in the cells.^31^ Here we showed that CPNE5 triggered FAS degradation in the early secretory pathway, which would contribute to depleting intracellular FAS before it is transported to the cell membrane. At the same time, our WB results also showed that the overexpression of CPNE5 inhibited the phosphorylation of p65 in the NF-κB signaling pathway, and knockdown of CPNE5 led to an increase in the phosphorylation of p65 in the NF-κB signaling pathway (Figure S5). This may be part of the mechanism by which CPNE5 inhibits cardiac fibrosis, and further tests are needed to determine whether CPNE5 affects inflammatory cell infiltration and fibroblast activation.

The *in vivo* experiments also showed that the cardiomyocytes-specific overexpression of CPNE5 can improve cardiac dysfunction in a mouse heart failure model and inhibit cardiomyocyte apoptosis by reducing the expression of FAS protein. In addition, we found that CPNE5 could improve FAS mediated cardiac injury. Similarly, systemic CPNE5 knockout mice showed reduced cardiac function and increased cardiomyocytes apoptosis. However, we cannot rule out the effect of other cells, such as inflammatory cells and endothelial cells, on cardiac function in systemic CPNE5 knockout mice, which needs to be verified by myocardial cell-specific knockout mice. Clarifying the expression pattern of CPNE5 in different types of cells and its specific effect on the FAS signaling pathway in the whole knockout mice will contribute to a comprehensive understanding of its role in apoptosis.

In conclusion, our findings suggest that CPNE5 plays a crucial role in modulating cardiac responses to stress and injury. The interaction between CPNE5 and FAS provides a mechanism by which CPNE5 can influence apoptosis and cardiac function. However, it remains unclear whether CPNE5 have an effect on contractile function and ion channels in cardiomyocytes. Further studies are needed to elucidate the complete molecular mechanisms underlying the protective effects of CPNE5 and to explore the feasibility of targeting this pathway in the clinical setting. In addition, studying the role of CPNE5 in other models of cardiac stress and its interaction with other apoptosis related proteins could provide a more complete understanding of the function of CPNE5.

### Limitations of the study

Although in this study we have elucidated part of the mechanism by which CPNE5 exerts a protective role in the heart during heart failure, we lack clinical experimental evidence. The next step could involve collecting human heart failure samples to clarify the changes in CPNE5 and validating its protective effects in human pluripotent stem cell-derived cardiomyocytes.

It is worth noting that the use of genetically modified myocyte-conditional knockout mice might further deepen the mechanistic insights of our study. However, due to the significant limitation of the current laboratory techniques needed to generate cardiomyocyte-specific *Cpne5*-knockout mouse models, we are unable to incorporate this critical experiment in this study, and we will perform related experiments when the laboratory technique is established.

## Resource availability

### Lead contact

Further information and requests for resources and reagents should be directed to and will be fulfilled by the lead contact, Prof. Yigang Li (liyigang@xinhuamed.com.cn).

### Materials availability

This study did not generate any new unique reagents.

### Data and code availability

• All data reported in this study (including raw data) are available from the lead contact upon request.
• No new code was developed for this article.
• Any additional information required to study the data presented in this article is available from the lead contact upon request.

## Acknowledgments

This work was supported by the grants from the State Key Program of 10.13039/501100001809National Natural Science Foundation of China (grant number: 82130009 to Y.-G.L.), the National Natural Science Foundation of China (grant numbers: 82070515 to Y.-G.L.), Shanghai Leading Talent Plan 2020 (Y.-G.L.), Shanghai City Committee of Science and Technology Research Projects (grant numbers: 19411963500 and 201409005600 to Y.-G.L.), Shanghai Arrhythmia Research Center Project (2022ZZ01008 to Y.-G.L.), the National Natural Science Foundation of China (grant number: 82100389 to Y.-D.F.).

## Author contributions

T.Z. and Y.B. contributed equally. T.Z. and Y.B. designed and performed the experiments, completed statistical analysis, and drafted the article. Y.F., Z.W., P.Y., Q.C., and Y.Z. helped to perform the immunohistochemical study. J.Y., K.C., Z.W., J.Q., and W.L. helped to perform the molecular biology studies. Y.W. helped to revise the methods. Q.W. and Y.L. conceived the project and edited the article.

## Declaration of interests

The authors declare no competing interests.

## STAR★Methods

### Key resources table

**Table:** 

REAGENT or RESOURCE	SOURCE	IDENTIFIER
**Antibodies**		

anti-CPNE5	Proteintech	Cat# 18097-1-AP; RRID:AB_2878499
anti-FAS	Abcam	Cat# ab271016; RRID: AB_3712731
anti-ANP	ABclonal	Cat# A14755; RRID: AB_2761631
anti-BNP	ABclonal	Cat# A23996; RRID: AB_3712732
anti-β-actin	ABclonal	Cat# AC038; RRID: AB_2863784
anti-Flag	Proteintech	Cat# 66008-4-Ig; RRID: AB_2918475
anti-Cleaved Caspase3	Cell Signaling Technology	Cat# 9661; RRID: AB_2341188
anti-Cleaved Caspase8	Cell Signaling Technology	Cat# 8592; RRID: AB_10891784
anti-FADD	Proteintech	Cat# 14906-1-AP; RRID: AB_2100486
anti-SEC13	Proteintech	Cat# 15397-1-AP; RRID: AB_2186234
anti-LC3	Proteintech	Cat# 14600-1-AP; RRID: AB_2137737
anti-Puromycin	KeraFASt	Cat# EQ0001; RRID: AB_2620162
anti-p-eiF2α	Cell Signaling Technology	Cat# 3398; RRID: AB_2096481
anti-SQSTM1/P62	Cell Signaling Technology	Cat# 5114; RRID: AB_10624872
anti-CALCOCO1	Proteintech	Cat# 19843-1-AP; RRID: AB_10637265
anti-cardiac troponin T	Proteintech	Cat# 68300-1-Ig; RRID: AB_3085051
anti-CPNE5	Novus	Cat# NBP1-84406; RRID: AB_11013078
anti-ERp72/ER marker	Proteintech	Cat# 14712-1-AP; RRID: AB_2160973
anti-LAMP1/Lysosome marker	Santa Cruz	Cat# sc-20011; RRID: AB_626853

**Bacterial and virus strains**		

AAV9-OE-*Cpne5*	Hanheng Biotechnology Co., Ltd. (Shanghai, China)	N/A
AAV9-OE-*Fas*	Hanheng Biotechnology Co., Ltd. (Shanghai, China)	N/A

**Biological samples**		

heart tissue (mus)	This paper	N/A

**Chemicals, peptides, and recombinant proteins**		

collagenase II	Worthington	LS004176
Opti-MEM™ Medium	Thermo Fisher Scientific	31985070
Lipofectamine™3000	Thermo Fisher Scientific	L3000-015
CHX	MCE	HY-12320
Chloroquine	MCE	HY-17589A
MG132	Selleck Chemicals	S2619
FASL protein	MCE	HY-P74157
puromycin 2HCl	Selleck Chemicals	S7417
phosphatase inhibitors	Epizyme	GRF101
protease inhibitors	Epizyme	GRF102
IP lysis buffer	Thermo Fisher Scientific	87787
Protein A/G magnetic beads	MCE	HY-K0202
Trizol Reagent	Thermo Fisher Scientific	15596026
Prime Script™ RT Master Mix Kit	Takara	RR036A

**Experimental models: Organisms/strains**		

Mouse: Male CPNE5 knockout (KO) mice	Cyagen Biosciences Biotechnology Co., Ltd.(Suzhou, China)	N/A

**Oligonucleotides**		

mouse CPNE5 siRNA sequence: Sense 5’-GCACCGAGGUCAUAGAUAATT-3’; Antisense 5’-UGUUUAGAU AGAUCCGGGCTT-3’.	Genepharma Biotechnology Co., Ltd. (Jiangsu, China)	N/A
mouse FAS siRNA sequence: Sense 5’-AAGAAGAAGUUCACAGAAATT-3’; Antisense 5’-UUUCUGUGAA CUUCUUCUUTT-3’.	Genepharma Biotechnology Co., Ltd. (Jiangsu, China)	N/A
mouse CPNE5 forward primer 5’-ATGTTGACTCCAAGAGCCCG-3’ mouse CPNE5 reverse primer 5’-CATCCAAAGACCACTTCCGTT-3’	This paper	N/A
mouse FAS forward primer 5’-GCTTGCTGGCTCACAGTTAAG-3’ mouse FAS reverse primer 5’-GCTCAGCCTAGTTTTCAGGTT-3’	This paper	N/A
mouse β-actin forward primer 5’-TAGGCACCAGGGTGTGATGG-3’ mouse β-actin reverse primer 5’-CATGGCTGGGGTGTTGAAGG-3’	This paper	N/A

**Software and algorithms**		

ImageJ software (version 4.0)	National Institutes of Health	https://imagej.net/ij/.
GraphPad Software	Graphpad Software Inc.	https://www.graphpad.com/scientific-software/prism/

### Experimental model and study participant details

#### Animal study

The describe research involve animal experiments that were approved by the institutional animal care and use committee of Xin Hua Hospital, Shanghai Jiaotong University. All animal experiments were consistent with the Guide for the Care and Use of Laboratory Animals published by the U.S. National Institutes of Health. To generate CPNE5 knockout (KO) mice, the CRISPR/Cas9 system was utilized. Specifically, sgRNA targeting a sequence in exon 2 of the mouse CPNE5 gene and Cas9 mRNA were injected into one-cell embryos to produce offspring with the desired mutation. Genotyping of the CPNE5 KO mice was performed using specific primers, and the genotype was further verified through taking cDNA from the mouse tails.

### Method details

#### Plasmid, small RNA interference, and adeno-associated virus (AAV)

The cDNA of mouse CPNE5 or FAS tagged with flag or ha was ligated with pcDNA3.1 (+) vector to construct plasmids. siRNAs and negative controls were designed and synthesized by Genepharma Biotechnology Co., Ltd. (Jiangsu, China). NC siRNA was employed as a negative control under similar conditions. siRNAs used in our experiment were:

mouse CPNE5 siRNA sequence: Sense 5’-GCACCGAGGUCAUAGAUAATT-3’; Antisense 5’-UGUUUAGAUAGAUCCGGGCTT-3’.

mouse FAS siRNA sequence: Sense 5’-AAGAAGAAGUUCACAGAAATT-3’; Antisense 5’-UUUCUGUGAACUUCUUCUUTT-3’.

For AAV9 expressing of CPNE5 and FAS overexpression in mice, the entire coding region of the mouse CPNE5 or FAS cDNA fusion express flag or ha tags were subcloned into the pHBAAV9-cTNT-MCS vector downstream of the cardiac troponin-T promoter. Male mice were given adenovirus (5.0 × 10^11^ V.g /mouse) using tail intravenous injection. The corresponding control mice received pHBAAV9-cTNT-Vector injections at the same dose respectively.

#### Echocardiography and surgical procedures

Echocardiography was used to assess cardiac function, mice were anesthetized by inhalation of 2% isoflurane and scanned using the VisualSonics Vevo 2100 imaging system. Ventricular dimensions were averaged from 3 to 5 cardiac cycles using M-mode imaging. Ejection fraction (EF) was calculated by the formula (LVEDV-LVESV)/LVEDV∗100%. To induce cardiac pressure overload in male mice, transverse aortic constriction (TAC) was performed. A 7–0 nylon suture was used to tie a ligature around a 28-gauge needle to create an aortic constriction. Sham operated mice underwent the same surgical procedure but without aortic constriction. Myocardial ischemia-reperfusion (I/R) injury in male mice is conducted by ligate of the left anterior descending artery for 30 minutes, followed by the release of the slip knot to allow reperfusion for 1 week. In this study, both ischemia-reperfusion (I/R) and transverse aortic constriction (TAC) models are used to comprehensively evaluate CPNE5's role in both acute and chronic cardiac stress settings aligning with prior studies investigating conserved molecular pathways across diverse cardiac injuries.^32^^,^^33^ For both the TAC and I/R surgical procedures, mice were anesthetized with a mask and inhaled 2% isoflurane and euthanized after echocardiography by swift decapitation according to NIH guidelines.

#### Cardiomyocytes isolation and transfection

Briefly, hearts from 1-day-old mice were finely chopped and then digested using collagenase II (Worthington, cat. no. LS004176) for 3 minutes at 37°C. After digestion, the collected cells were incubated in fresh culture medium, which consisted of DMEM supplemented with 20% fetal calf serum (FBS). This incubation was carried out at 37°C.

And 5% CO_2_ for 90 minutes, allowing for the separation of cardiac fibroblasts from the cardiomyocytes. After 48 hours of cultivation, the cardiomyocytes exhibited rhythmic pulsations, indicating their suitability for subsequent experimental procedures. Then transfection was carried out using 3 ug of plasmid. This was achieved by mixing it with Opti-MEM™ Medium (Thermo Fisher Scientific, cat. no. 31985070), which contained 3 μL of Lipofectamine™3000 and 3 μL of P3000 reagent (Thermo Fisher Scientific, cat. no. L3000-015). Additionally, 50 nM of siRNA were transfected using Opti-MEM™ Medium with 3 μL of Lipofectamine RNAiMAX (Thermo Fisher Scientific, cat. no. 13778100, ). Following this transfection step, the cells were added with 5μM CHX (MCE, cat. no. HY-12320) for protein stability study, 1 μM Chloroquine (MCE, cat. no. HY-17589A) for lysosome inhibition study, 4 μM MG132 (Selleck Chemicals, cat. no. S2619) for proteasome inhibition study, 500ng/ml FASL protein (MCE, cat. no. HY-P74157) to activate the FAS receptor, 5 μM puromycin 2HCl (Selleck Chemicals, cat. no. S7417) for protein synthesis study.

#### Western blotting

Equal amounts (typically 30 or 50 ug) of cellular or tissue proteins were separated on SDS-PAGE gels. Following this, the proteins were transferred onto a PVDF membrane. The membranes were then blocked with 5% skim milk for one hour at room temperature and incubated with the specified primary antibodies at 4°C overnight. On the following day, the PVDF membrane was washed with TBST and incubated with an HRP-conjugated secondary antibody at room temperature for one hour. Subsequently, the signal on the membrane, which had been activated by the chemiluminescence (ECL) reagent, was detected using an ECL system. Antibodies used were anti-CPNE5 (1:1000, Proteintech, cat. no. 18097-1-AP), anti-FAS (1:1000, Abcam, cat. no. ab271016), anti-ANP (1:1000, ABclonal, cat. no. A14755), anti-BNP (1:1000, ABclonal, cat. no. A23996), anti-β-actin (1:1000, ABclonal, cat. no. AC038), anti-Flag (1:1000, Proteintech, cat. no. 66008-4-Ig), anti-Cleaved Caspase3 (1:1000, Cell Signaling Technology, cat. no. 9661), anti-Cleaved Caspase8 (1:1000, Cell Signaling Technology, cat. no. 8592), anti-FADD (1:1000, Proteintech, cat. no. 14906-1-AP), anti-SEC13 (1:1000, Proteintech, cat. no. 15397-1-AP), anti-LC3 (1:1000, Proteintech, cat. no. 14600-1 AP), anti-Puromycin (1:1000, KeraFASt, cat. no. EQ0001), anti-p-eiF2α (1:1000, Cell Signaling Technology, cat. no. 3398), anti-SQSTM1/P62 (1:1000, Cell Signaling Technology, cat. no. 5114), anti-CALCOCO1 (1:1000, Proteintech, cat. no. 19843-1-AP).

#### Immunoprecipitation (IP) assay

Cells were lysed utilizing 500 μl IP lysis buffer (Thermo Fisher Scientific, cat. no. 87787) with phosphatase inhibitors (epizyme, cat. no. GRF101) and protease inhibitors (epizyme, cat. no. GRF102). Subsequently, the lysates were gathered and subjected to centrifugation. Following the centrifugation process, the protein concentration was determined using a BCA protein quantification kit, following the manufacturer's guidelines meticulously. Cell lysates containing the same amount of protein were either used as whole cell lysate (WCL) or co-immunoprecipitated with Protein A/G magnetic beads (MCE, cat. no. HY-K0202) coated with specific antibodies at 4°C for 2 h.

#### IP-MS

After transfection of NMCMs with Flag-tagged CPNE5, immunoprecipitation (IP) was carried out utilizing an anti-Flag primary antibody. The precipitates obtained were separated by SDS-PAGE gels and subsequently stained with Coomassie blue. Bands displaying differences between the Flag-*Cpne5* and control groups were further analyzed by liquid chromatography-electrospray ionization-mass spectrometry (LC-ESI-MS), conducted at Shanghai applied protein technology co. ltd in Shanghai, China.

#### Immunofluorescence and histological analysis

Cultured NMCMs were fixed using 4% paraformaldehyde (PFA) for 15 minutes at room temperature. Subsequent to being rinsed three times with phosphate-buffered saline (PBS), the samples were permeabilized with 0.5% Triton X-100 in PBS for 10 minutes. This was followed by blocking the samples with 5% goat serum for an hour at room temperature. Thereafter, the cells were incubated with primary antibodies overnight at 4°C. After three washes with PBST, the samples were incubated with the corresponding secondary antibodies for an hour at room temperature, and the nuclei were subsequently stained with DAPI for 5 minutes. Mouse hearts were immobilized in a solution of 4% PFA and subsequently dehydrated for embedding in paraffin. To detect fibrosis, Masson’s trichrome staining was applied to the paraffin sections. The quantity of blue collagen staining was then assessed utilizing software ImageJ. In order to perform tissue immunofluorescence experiments, the slides were first deparaffinized with xylene and then dehydrated through a graded series of ethanol concentrations. Antigen retrieval was achieved by microwaving the slides in citrate solution for 10 minutes. Subsequently, nonspecific binding sites were blocked with 5% goat serum. The slides were then incubated with primary antibodies at 4°C overnight. The following day, they were washed three times with PBST and incubated with the corresponding secondary antibodies for 1 hour at room temperature. The nuclei were stained with DAPI for 5 minutes. In order to detect apoptosis of cells and tissue slides, terminal dUTP nick end labeling (TUNEL) staining was followed by secondary antibody staining for 30 minutes at room temperature. Images were captured by confocal microscopy (Leica TCS SP8) or fluorescence microscope (Olympus BX51). The intensity profiles were analyzed by “plot proile” module in ImageJ.

The primary antibodies used in this study for immunofluorescence included anti-cardiac troponin T (cTnT) (1:200, Proteintech, cat. no. 68300-1-Ig), anti-CPNE5 (1:100, Novus, cat. no. NBP1-84406), anti-ERp72/ER marker (1:100, Proteintech, 14712-1-AP), anti-LAMP1/Lysosome marker (1:100, Santa Cruz, cat. no. sc-20011).

#### RT-qPCR

Heart tissues or cells were used to extract total RNA using Trizol Reagent (Thermo Fisher Scientific, cat. no. 15596026). The Prime Script™ RT Master Mix Kit (Takara, cat. no. RR036A) was employed to synthesize first-strand cDNA from the extracted total RNA. The expression levels of the targeted genes were normalized to the expression of β-actin, which served as an endogenous internal control.

Primer sequences were as follows:

mouse CPNE5 forward primer 5’-ATGTTGACTCCAAGAGCCCG-3’

mouse CPNE5 reverse primer 5’-CATCCAAAGACCACTTCCGTT-3’

mouse FAS forward primer 5’-GCTTGCTGGCTCACAGTTAAG-3’

mouse FAS reverse primer 5’-GCTCAGCCTAGTTTTCAGGTT-3’

mouse β-actin forward primer 5’-TAGGCACCAGGGTGTGATGG-3’

mouse β-actin reverse primer 5’-CATGGCTGGGGTGTTGAAGG-3’

### Quantification and statistical analysis

All the statistical data were presented as the mean±SD. Statistical analyses were performed by GraphPad Prism v.8.0 and P < 0.05 was considered statistically significant. Unpaired two-tailed Student’s t-test (two groups), oneway ANOVA with Dunnett’s posthoc test (more than two groups) were used to determine statistical significance of experimental data.

## Supplemental information

Document S1. Figures S1–S8

Document S2. Full unedited gel images

Data S1. RT-qPCR
