# Supplementary material for: CPNE5 overexpression inhibits cardiomyocytes apoptosis by promoting the degradation of FAS receptor
Source: iScience. 2025 Aug 6;28(9):113302. doi: 10.1016/j.isci.2025.113302 (PMC12398889; doi:10.1016/j.isci.2025.113302)
Supplement: Document S1. Figures S1–S8 [file mmc1.pdf]

iScience, Volume *28*

Supplemental information

CPNE5 overexpression inhibits cardiomyocytes apoptosis by promoting the degradation of FAS receptor

Tingting Zhao, Yangjinming Bai, Yudong Fei, Zhixing Wei, Pengcheng Yao, Qianji Che, Yichao Zhang, Ji Yan, Kaiyan Chen, Zhengyang Wu, Junhao Qiu, Yuepeng Wang, Wei Li, Qian Wang, and Yigang Li

Supplemental figures and figures legend

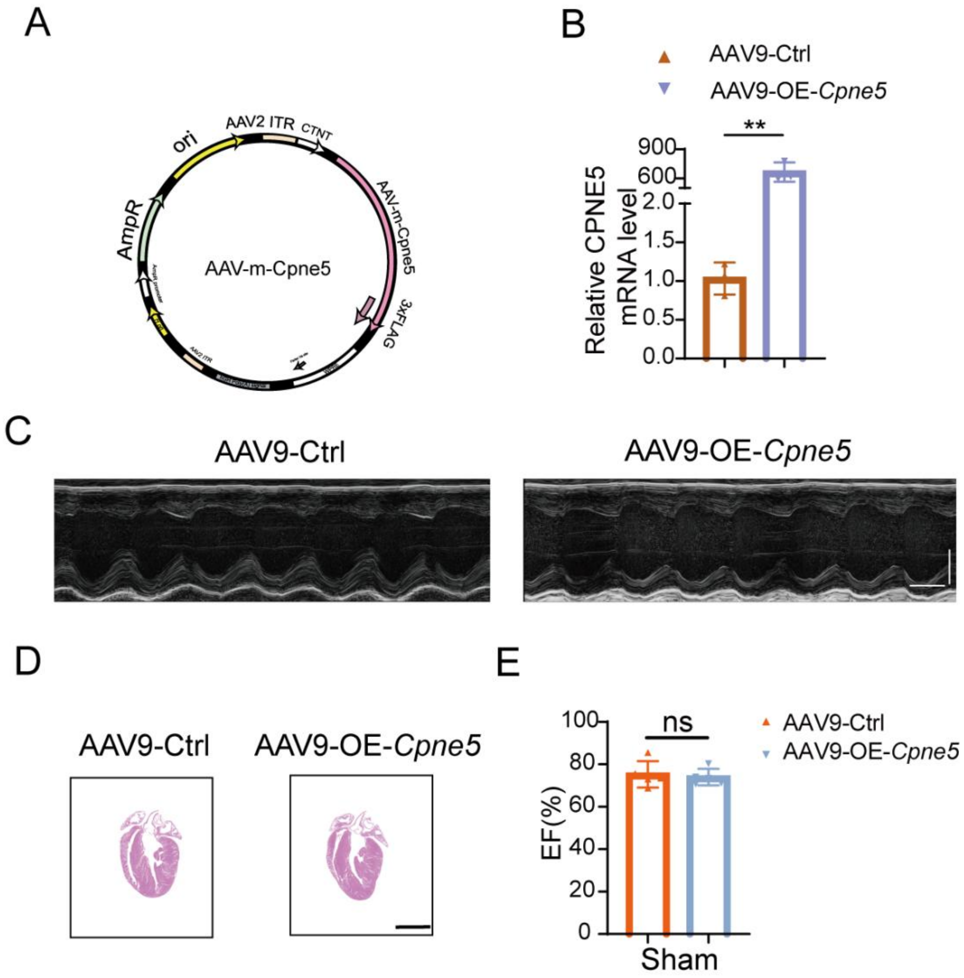

Figure S1. The construction strategy of myocardial-specific CPNE5 overexpression virus and verify the overexpression efficiency, ralated to Figure 2

(A) Construction strategies for cardiomyocytes-specific overexpression of CPNE5.

(B) Quantification CPNE5 mRNA level of in overexpression CPNE5 mice, normalized to β-actin (n=3).

(C) Representative M-mode echocardiographic recordings from sham-operated AAV9-Ctrl and AAV9-OE-*Cpne5* mice, horizontal bar=100 ms, vertical bar=2 mm.

(D) Histological sections stained with H&E (bar=2 mm) from sham-operated AAV9-Ctrl and AAV9-OE-*Cpne5* mice.

(E) Quantitative analyses of echocardiographic measurements of sham-operated AAV9-Ctrl and AAV9-OE-*Cpne5* mice (n=5). All data are presented as mean±SD. **, P < 0.01; ns, not significantly different.

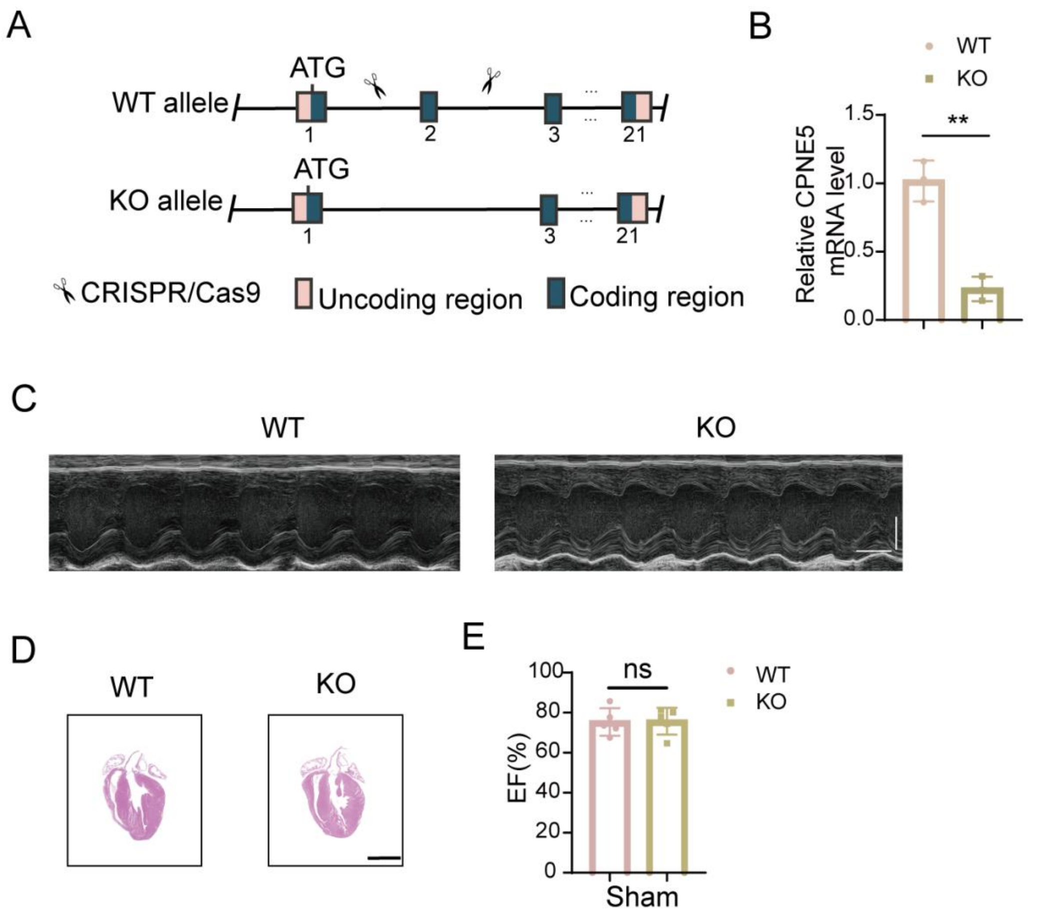

Figure S2. Construction strategy and validation of knockdown efficiency in whole-body CPNE5 knockout mice, ralated to Figure 3

(A) Construction strategies for whole body knockout of CPNE5 (KO) mice.

(B) Quantification CPNE5 mRNA level of in KO mice, normalized to β-actin (n=3).

(C) Representative M-mode echocardiographic recordings from sham-operated WT and KO mice, horizontal bar=100 ms, vertical bar=2 mm.

(D) Histological sections stained with H&E (bar=2 mm) from sham-operated WT and KO mice.

(E) Quantitative analyses of echocardiographic measurements of sham-operated WT and KO mice (n=5). All data are presented as mean±SD. **, $P < 0.01$; ns, not significantly different.

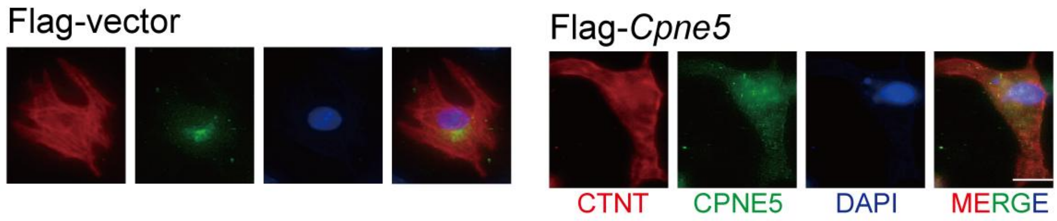

Figure S3. Distribution of CPNE5 in NMCMs after overexpression, ralated to Figure 4
Immunofluorescence revealed CPNE5 (green) intracellular distribution in NMCMs (red), the nucleus is blue, bar=20 μm.

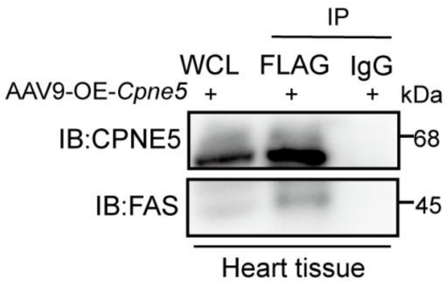

Figure S4. CPNE5 interact with FAS in CPNE5 overexpressing heart tissues, ralated to Figure 4
IP analysis with anti-FLAG and IB with anti-bodies of CPNE5 and FAS, respectively, in overexpression CPNE5 mice heart tissue. WCL: whole cell lysates. IgG was used as a negative control to rule out nonspecific interactions.

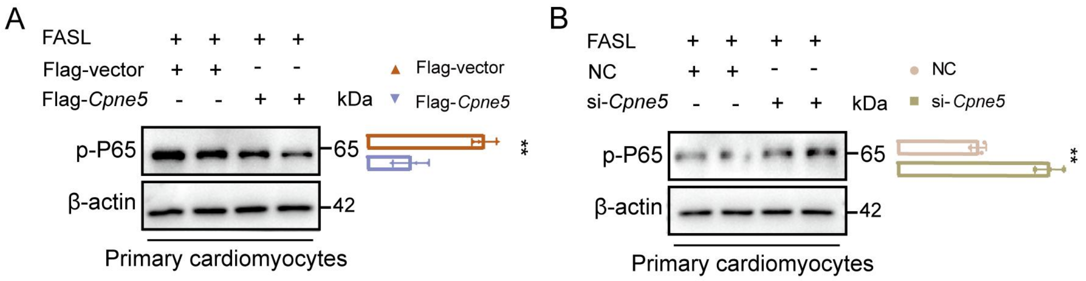

Figure S5. Overexpression of CPNE5 inhibit the phosphorylation of P65, ralated to discussion
(A and B) After treated with FASL protein, NMCMs overexpression CPNE5 (A) or knockdown CPNE5 group (B) were detected p-P65 protein level by western blot. Bar graph on the right quantification protein level of (A) and (B), normalized to β-actin (n=3). All data are presented as mean±SD. **, P < 0.01.

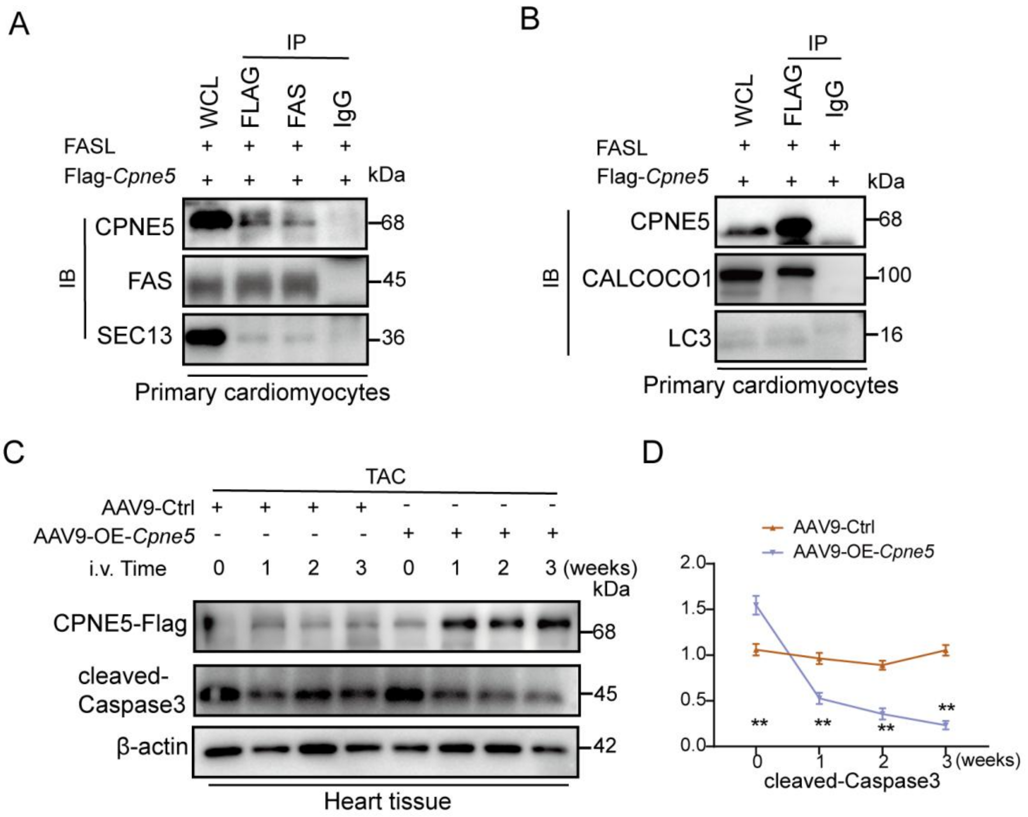

Figure S6. FASL did not affect the interaction between CPNE5 and FAS, ralated to Figure 5

(A) Following FASL induction, transfected NMCMs expressing Flag-CPNE5 were subjected to immunoprecipitation (IP) with anti-FLAG and anti-FAS antibodies. Immunoblotting (IB) was then performed with antibodies against CPNE5, FAS, SEC13. WCL: whole cell lysates. IgG was used as a negative control to rule out nonspecific interactions.

(B) Following FASL induction, transfected NMCMs expressing Flag-CPNE5 were subjected to immunoprecipitation (IP) with anti-FLAG antibody. Immunoblotting (IB) was then performed with antibodies against CPNE5, CALCOCO1 and LC3. WCL: whole cell lysates. IgG was used as a negative control to rule out nonspecific interactions.

(C and D) After intravenous tail vein (i.v.) injection of AAV9-OE-*Cpne5* at equal doses, we established TAC models at 0, 1, 2, and 3 weeks post-injection (at each time point n = 3). Western blot analysis was performed to assess CPNE5 expression levels and Caspase3 activation. All data are presented as mean±SD. **, $P < 0.01$; *, $P < 0.05$; ns, not significantly different.

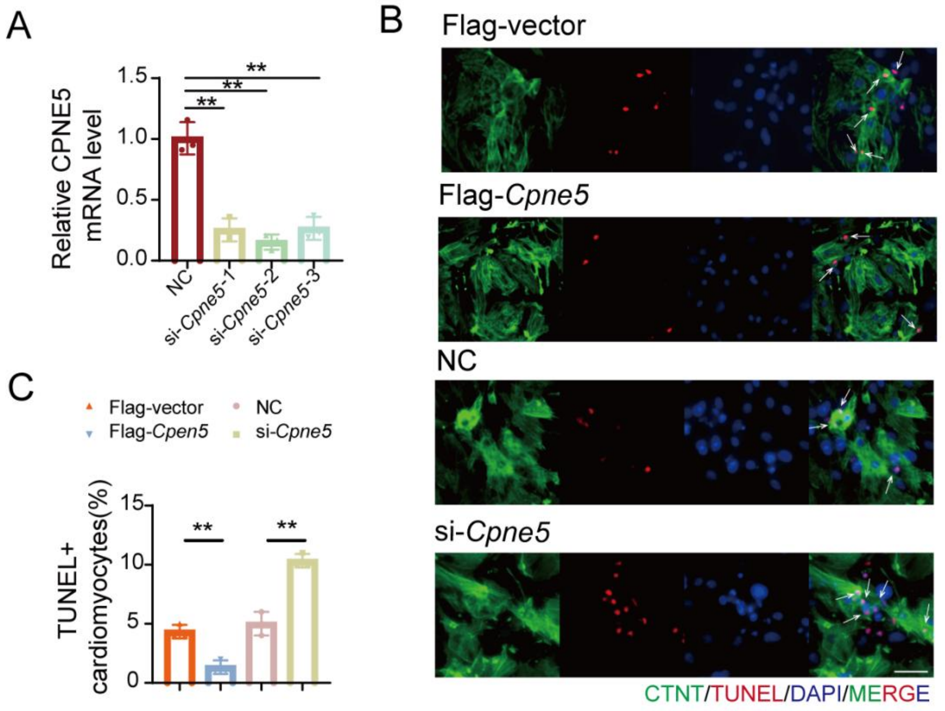

Figure S7. Validation of CPNE5 mRNA knockdown efficiency by three siRNA sequences, ralated to Figure 6

(A) Quantification CPNE5 mRNA level of in group NC, si-*Cpne5*-1, si-*Cpne5*-2, si-*Cpne5*-3, normalized to β-actin (n=3). si-*Cpne5*-1, the interference sequence used in Figure 6A, 6D, 8H and 8J of the manuscrip, is documented in the methods section.

si-*Cpne5*-2 sequences: Sense 5'-GCCCGGAUCUAUCUAAACATT-3'; Antisense 5 -UGUUUAGAUAGAUCCGGGCTT-3'.

si-*Cpne5*-3 sequences: Sense 5'-GAGAGAUCAUUCAGCAUUATT-3'; Antisense 5'-UAAUGCUGAAUGAUCUCUCTT-3'.

(B) Representative image showing immunofluorescence of TUNEL (red, arrows), DAPI (blue, for nuclei), and cardiac troponin (green; bar=50 μm) in overexpression (Flag-*Cpne5*) or knockdown (si-*Cpne5*) CPNE5 NMCMs under basal conditions. Flag-vector: empty vectors, NC: negative control.

(C) Statistical analysis of TUNEL-positive cells in (B). All data are presented as mean±SD. **, $P < 0.01$.

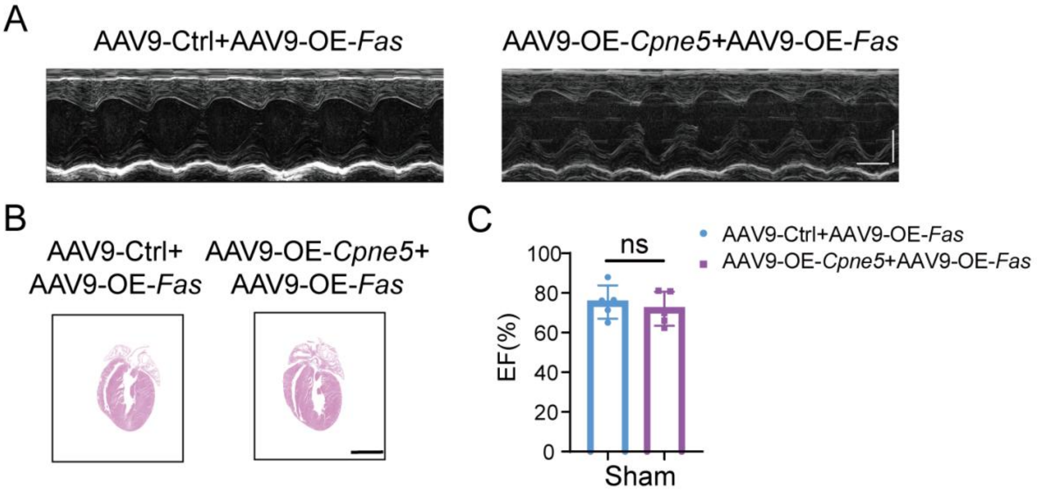

Figure S8. Increasing FAS expression specifically in the heart at baseline did not affect cardiac function or morphology in mice, ralated to Figure 7.

(A) Representative M-mode echocardiographic recordings from sham-operated AAV9-Ctrl+AAV9-OE-*Fas* and AAV9-OE-*Cpne5*+AAV9-OE-*Fas* mice, horizontal bar=100 ms, vertical bar=2 mm.

(B) Histological sections stained with H&E (bar=2 mm) from sham-operated AAV9-Ctrl+AAV9-OE-*Fas* and AAV9-OE-*Cpne5*+AAV9-OE-*Fas* mice.

(C) Quantitative analyses of echocardiographic measurements of sham-operated AAV9-Ctrl+AAV9-OE-*Fas* and AAV9-OE-*Cpne5*+AAV9-OE-*Fas* mice (n=5). All data are presented as mean±SD. ns, not significantly different.
