# Supplementary material for: CPNE5 overexpression inhibits cardiomyocytes apoptosis by promoting the degradation of FAS receptor
Source: iScience. 2025 Aug 6;28(9):113302. doi: 10.1016/j.isci.2025.113302 (PMC12398889; doi:10.1016/j.isci.2025.113302)

Full unedited gel for Figure 1A

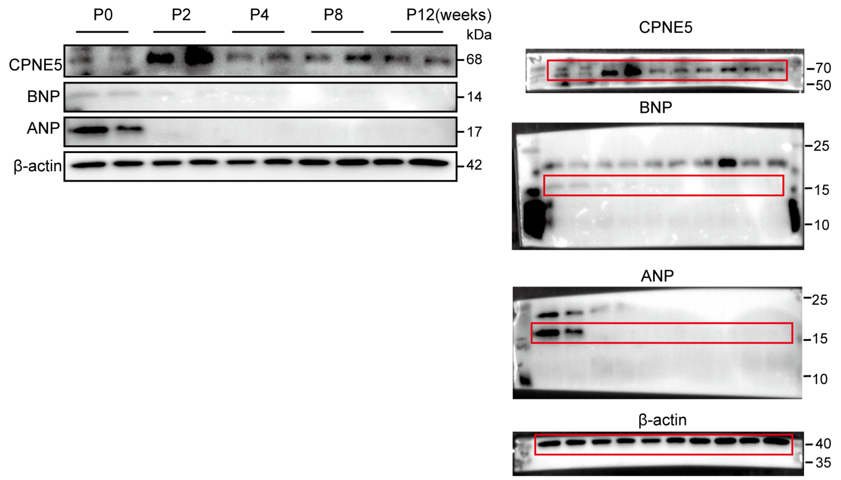

Full unedited gel for Figure 1B

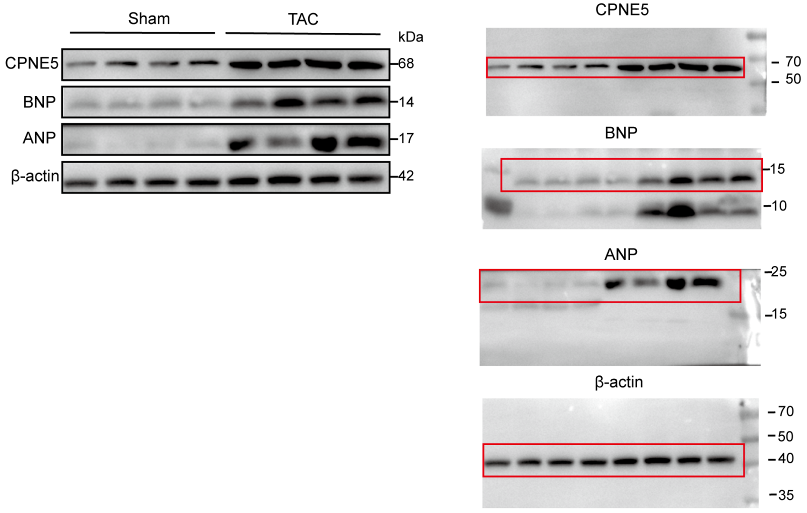

Full unedited gel for Figure 1C

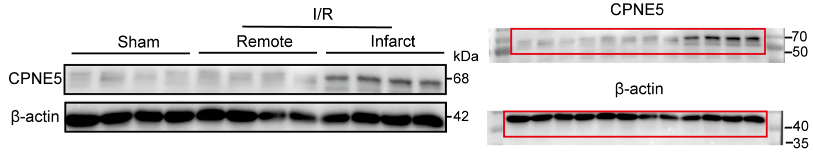

Full unedited gel for Figure 4B

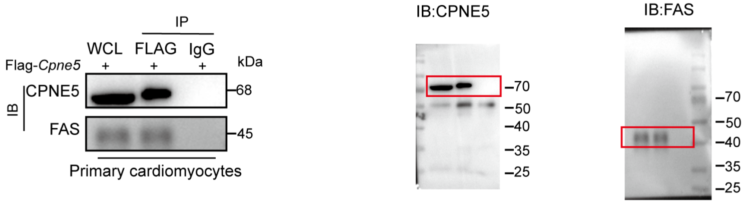

Full unedited gel for Figure 4E

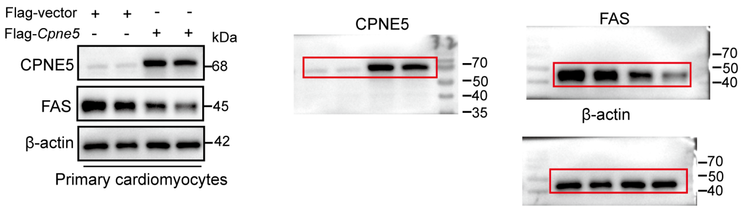

Full unedited gel for Figure 4J

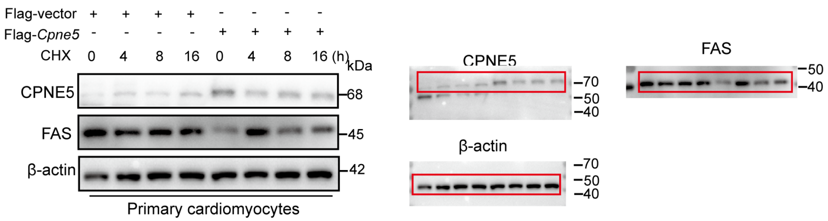

Full unedited gel for Figure 4L

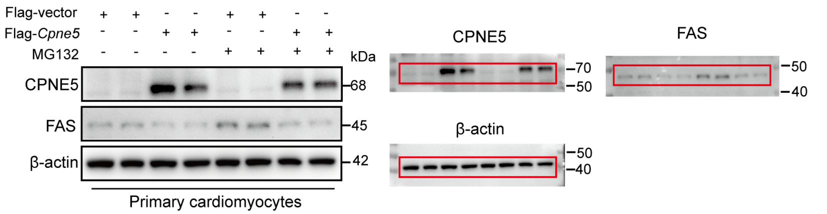

Full unedited gel for Figure 4N

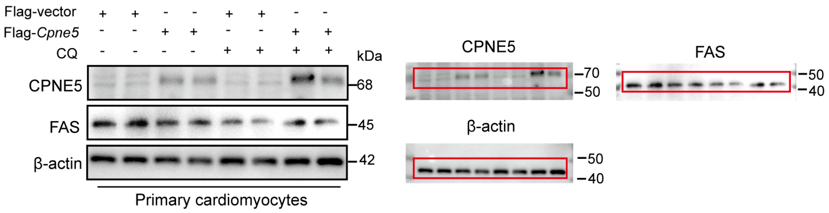

Full unedited gel for Figure 5B

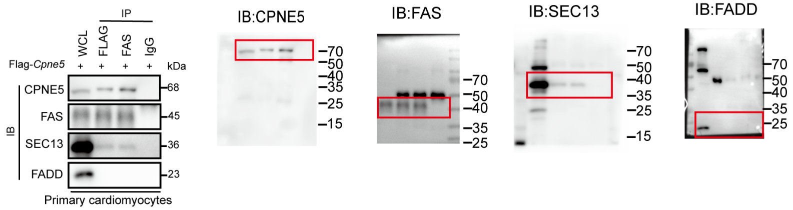

Full unedited gel for Figure 5G

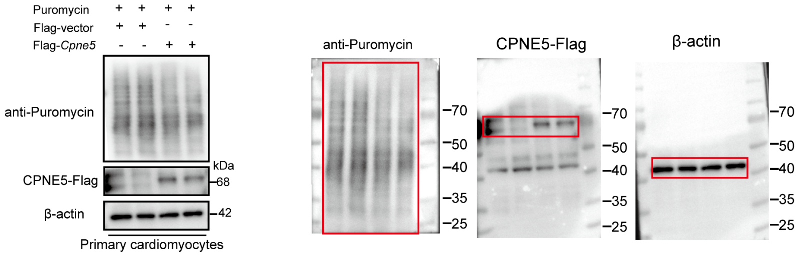

Full unedited gel for Figure 5H

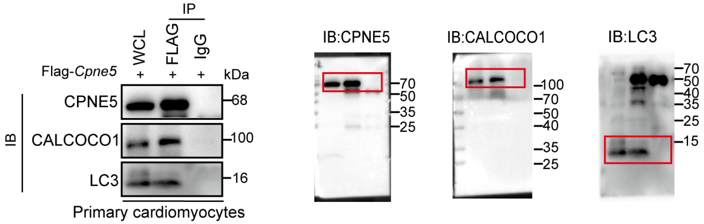

Full unedited gel for Figure 5I

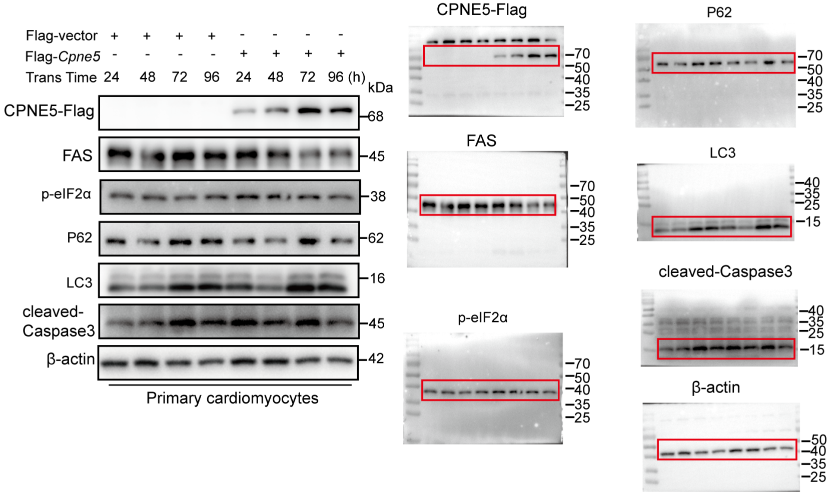

Full unedited gel for Figure 6C

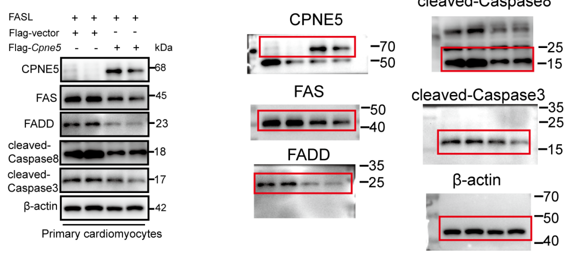

Full unedited gel for Figure 6D

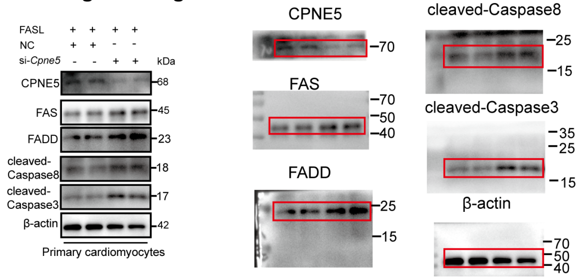

Full unedited gel for Figure 6E

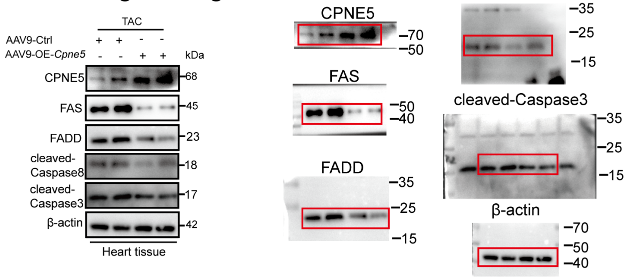

Full unedited gel for Figure 6F

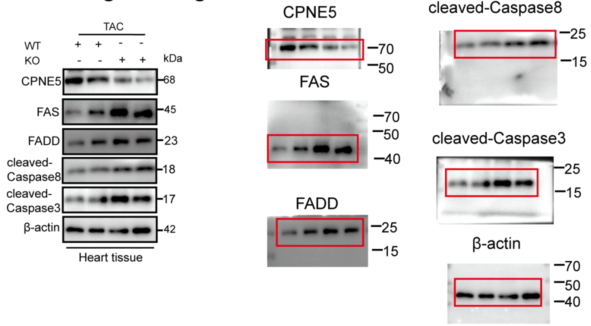

Full unedited gel for Figure 6G

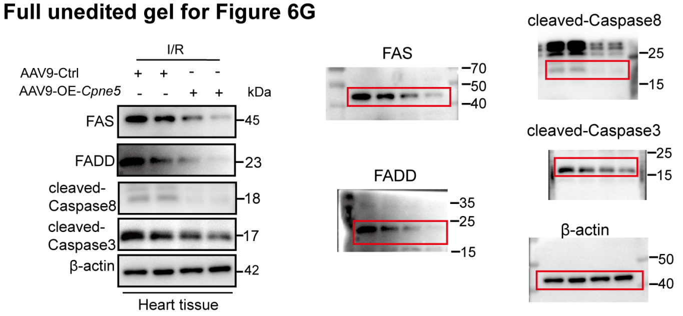

Full unedited gel for Figure 6H

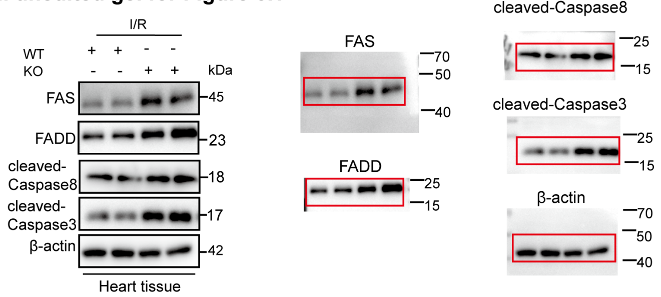

Full unedited gel for Figure 7H

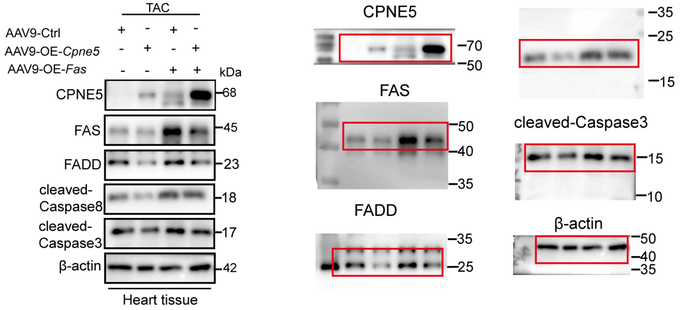

Full unedited gel for Figure 8C

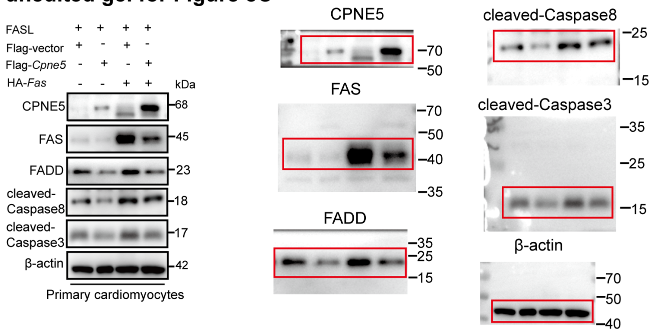

Full unedited gel for Figure 8J

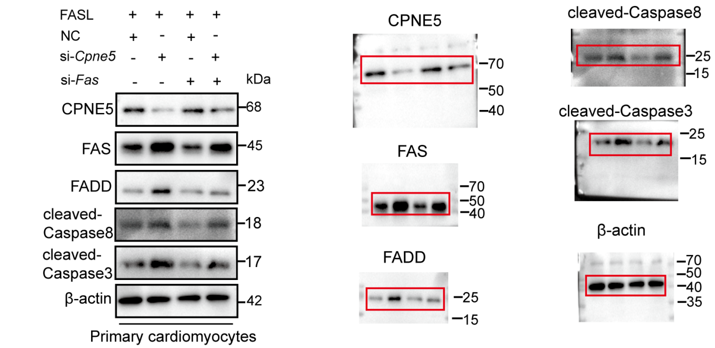

Full unedited gel for Figure S4A

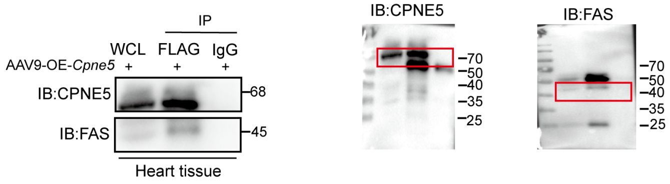

Full unedited gel for Figure S5A

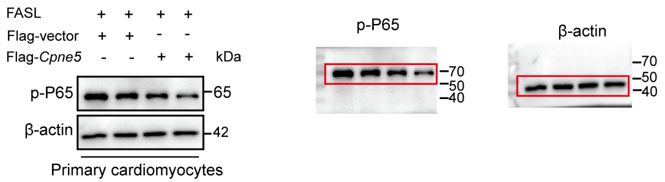

Full unedited gel for Figure S5B

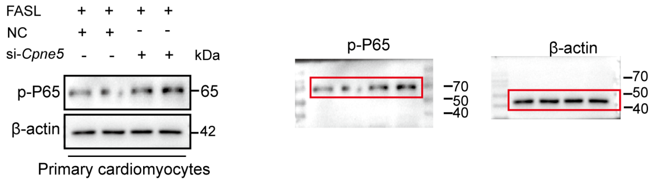

Full unedited gel for Figure S6A

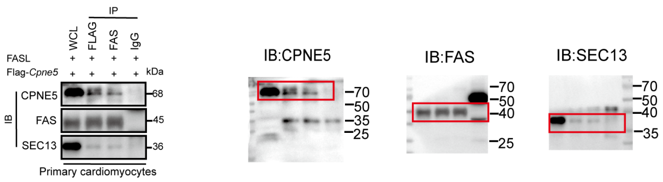

Full unedited gel for Figure S6B

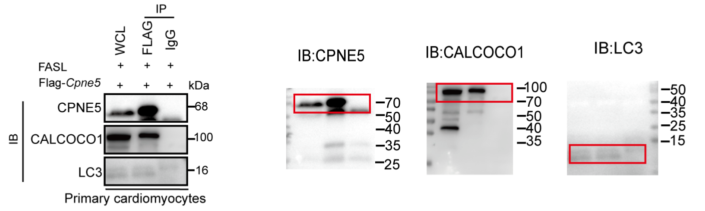

Full unedited gel for Figure S6C

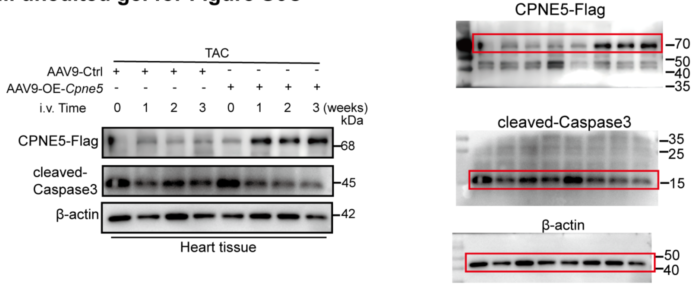

Supplement: Document S2. Full unedited gel images [file mmc2.pdf]
